# Monitoring cerebral oxygenation during balloon occlusion with multichannel NIRS

**DOI:** 10.1038/jcbfm.2013.207

**Published:** 2013-12-04

**Authors:** Christian Rummel, Christoph Zubler, Gerhard Schroth, Jan Gralla, Kety Hsieh, Eugenio Abela, Martinus Hauf, Niklaus Meier, Rajeev K Verma, Robert H Andres, Arto C Nirkko, Roland Wiest

**Affiliations:** 1 Support Center for Advanced Neuroimaging (SCAN), University Institute for Diagnostic and Interventional Neuroradiology, Inselspital, University of Bern, Bern, Switzerland; 2 Department of Neurology, Inselspital, University of Bern, Bern, Switzerland; 3 Department of Neurosurgery, Inselspital, University of Bern, Bern, Switzerland

**Keywords:** angiography, cerebral blood flow measurement, interventional neuroradiology, near infrared spectroscopy, neuroradiology

## Abstract

We report on oxygenation changes noninvasively recorded by multichannel continuous-wave near infrared spectroscopy (CW-NIRS) during endovascular neuroradiologic interventions requiring temporary balloon occlusion of arteries supplying the cerebral circulation. Digital subtraction angiography (DSA) provides reference data on the site, timing, and effectiveness of the flow stagnation as well as on the amount and direction of collateral circulation. This setting allows us to relate CW-NIRS findings to brain specific perfusion changes. We focused our analysis on the transition from normal perfusion to vessel occlusion, i.e., before hypoxia becomes clinically apparent. The localization of the maximal response correlated either with the core (occlusion of the middle cerebral artery) or with the watershed areas (occlusion of the internal carotid artery) of the respective vascular territories. In one patient with clinically and angiographically confirmed insufficient collateral flow during carotid artery occlusion, the total hemoglobin concentration became significantly asymmetric, with decreased values in the ipsilateral watershed area and contralaterally increased values. Multichannel CW-NIRS monitoring might serve as an objective and early predictive marker of critical perfusion changes during interventions—to prevent hypoxic damage of the brain. It also might provide valuable human reference data on oxygenation changes as they typically occur during acute stroke.

## Introduction

**Figure 1:**
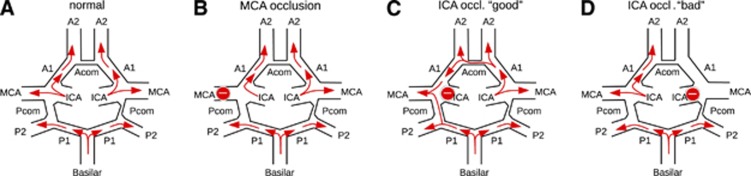
Schematic representation of different possible perfusion scenarios using the example of a patient with low vessel caliber in the Acom and the right Pcom. (**A**) Brain perfusion under normal conditions. (**B**) Occlusion of the left middle cerebral artery (MCA). As the occlusion site is distal to the circle of Willis (CoW), collateral flow could be expected only via usually not sufficient surface leptomeningeal collaterals through the watershed areas. Thus, the vascular core territory of the MCA will not be supplied adequately. (**C**) Occlusion of the left internal carotid artery (ICA). Collaterals via the Acom and left Pcom are sufficiently strong in this patient to maintain perfusion of all brain regions (‘good' collaterals, asymptomatic). (**D**) Occlusion of the right ICA. As the Acom and the right Pcom are not strong enough in this patient, in this situation the territories of the right ACA and MCA will not be supplied sufficiently (‘bad' collaterals, symptomatic).

Cerebro-vascular autoregulation, which is effected predominantly by the peripheral precapillary arterioles, attempts to maintain a constant blood flow of around 50 mL/100 g per minute (ref. 1) within a wide range of perfusion pressure. If cerebral arteries fail to provide sufficient perfusion pressure due to shunting within fistulas or steno-occlusive disease, then the subcortical and cortical collateral artery network has an important role in preserving blood and oxygen supply via numerous alternative pathways that maintain postocclusive perfusion pressure, thereby redistributing oxygenated blood to the deprived site. The most important collateral pathways encompass the circle of Willis (CoW) with the anterior and posterior communicating arteries (Acom, Pcom), see Figure 1, and pathways via retrograde collaterals of the ophthalmic and meningeal branches of the external carotid artery.

Balloon occlusion testing (BOT) by cerebral angiography is currently considered as a standard diagnostic test to evaluate the effectiveness of collateral circulation before therapeutic neurointerventional or surgical procedures that require (or carry the risk of) permanent occlusion of the internal carotid artery (ICA). Linskey *et al*^2^ showed a decreased risk of ischemic complications after BOT of 13%. However, false negative results may occur, since sufficient collateral circulation depends not only on anatomic variations and age, but also on arterial blood pressure and slow adaptation of middle sized vessel diameter, which may appear with delay after occlusion. In ∼80% of patients enrolled for carotid occlusion, brain perfusion of the affected hemisphere is supported by sufficient collaterals, whereas ∼20% of patients carry the risk of permanent ischemic brain injury as a consequence of carotid occlusion.^3^ Figure 1 summarizes different perfusion patterns that are observed during selective vessel occlusion (and as a consequence of spontaneous embolic stroke). Ischemic events or infarction may manifest as a consequence of permanent carotid occlusion even in the absence of any clinical signs and while stable blood flow and blood velocity measures are observed during occlusion.^4^ Since autoregulation may fail to maintain the cerebral perfusion reserve, ischemic events may occur after carotid occlusion depending on delayed variation of blood and local cerebral perfusion pressure.

The current standard technique for monitoring BOT, apart from assessing clinical tolerance, is continuous transcranial Doppler (TCD) sonography. The TCD monitoring is based on real-time measures of the blood velocities of the middle cerebral artery (MCA) during occlusion.^5^ Of note, blood velocity monitoring in the supplying arteries rather reflects territorial, not localized regional measures. Thus, hypoxia in watershed regions may remain undetected by TCD. Further, it may fail to yield interpretable results at all if no acoustic window is present.

Similar to TCD, near infrared spectroscopy (NIRS) may be considered as a bedside test that offers the potential to monitor cerebral concentrations of oxygenated and deoxygenated hemoglobin in real time. It provides a method for noninvasive and time-continuous assessment of tissue oxygenation^6, 7^ that is complementary to velocity or flow information from TCD or digital subtraction angiography (DSA). The latter mainly visualizes the degree of contrast inflow to arteries, the capillary bed and its subsequent outflow into venoles and veins. In BOT, an occlusion balloon is inflated for ∼20 minutes. During this time, repeated angiography of all brain supplying arteries is limited (‘snap shot' information) since exposure to radiation and iodinated contrast has to be minimized. In addition, depending on the used angiography system it is only possible to obtain information about the blood flow in monoplanar or biplanar projections. Often, this does not provide sufficient information about regional cerebral blood flow in many cortex regions. Angiography of the occluded vessel only informs about the flow stagnation at the occlusion balloon and does not reveal any information about tissue oxygenation of the subsequent territory. Therefore, the crucial information about the tissue at risk cannot be provided by angiography.

As functional magnetic resonance imaging (MRI), see e.g. Kim and Ogawa^8^ for review, NIRS information is fundamentally related to blood oxygenation, although the underlying mechanism is entirely different and also a different vasculature is interrogated. Employing the modified Beer–Lambert law^9^ continuous-wave (CW) NIRS estimates *relative changes* in oxy- and deoxyhemoglobin concentrations from measured changes in light attenuation in the wavelength range of 650 to 1,000 nm with subsecond temporal resolution. Because concentrations of both states of hemoglobin oxygenation can be assessed independently, CW-NIRS may provide an additional estimate for blood volume changes through total hemoglobin concentration.

Placement of near infrared light sources and detectors (optodes) on the scalp has lead to two established, mutually segregated approaches: (1) transcranial cerebral oxymetry and (2) functional NIRS. Transcranial cerebral oxymetry usually employs a small number (most often only two) of NIRS channels and often low-time resolution (several seconds) to estimate the cerebral oxygen saturation. In anesthesiology, frontal transcranial cerebral oxymetry is applied routinely to monitor interventions in critical patients, see e.g. ref. 10 for review. Functional NIRS, similar to functional MRI, takes advantage of the effects resulting from neurovascular coupling to relate observed absorbance changes to neuronal activity changes. Here, often multichannel settings are chosen since they cover large parts of the head and their temporal resolution is in the subsecond range. Functional NIRS has been applied in various fields of neuroscience over the last 15 years, see ref.^11^,^12^,^13^ for reviews.

During neuroangiographic interventions, percutaneous transluminal angioplasty (PTA) is needed for stenting of the ICA and requires short-term occlusion (within the range of seconds) of the artery. Certain embolic protection devices and BOT involve transient prolonged occlusion (within the range of minutes) of the ICA. The fact that these interventions take place under surveillance with DSA allows reliably assessing the effect of specific temporal and regional flow stagnation on hemoglobin concentration changes in NIRS monitoring. *Two-channel* transcranial cerebral oxymetry during various endovascular neuroangiographic interventions has been reported in refs. 14,15,16,17,18,19,20,21,22. Here, we report on retrospective analysis of interventions in nine patients monitored by *multichannel* CW-NIRS. We hypothesized that this method can be used to study whether sufficient collaterals allow the cerebral autoregulation to operate within a range of pressures that can be compensated, or whether it already operates at or beyond its limit due to a locally impaired perfusion pressure, thus effectively indicating ischemic penumbra as known in acute ischemic stroke.

## Materials and methods

### Patients

From a total of 35 patients that underwent neuroradiologic interventions with NIRS monitoring at our institution from July 2012 to July 2013, we selected those nine patients who received transient balloon occlusions of the cerebral arteries, see Table 1 for demographics and details of the endovascular interventions. The data were organized in three groups according to the occlusion site and duration: one patient underwent a series of MCA occlusions distal to the CoW during coil embolization of an aneurysm. The absence of sizable collaterals allows assessment of the ‘pure' effect of ischemia on multichannel CW-NIRS signals, see Figure 1B. Occlusions of ICA in a second group of patients were required during stent placement and performed proximal to the CoW (which could serve as main collateral, see Figure 1C). In the third patient group, prolonged occlusion was performed proximal to the CoW during BOT.

### Data Acquisition

#### Neuroangiological procedures

**Figure 2:**
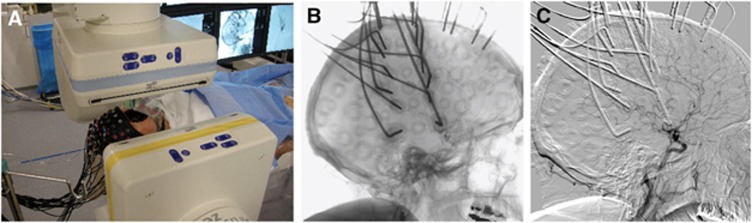
Near infrared spectroscopy (NIRS) measurement during neuroradiologic intervention. (**A**) Setting in the angiography suite. (**B**) Cerebral angiography with passage of a contrast agent bolus in patient L2. (**C**) Digital subtraction angiography of the same data as in panel **B**.

All interventions were performed on a biplane flat panel angiographic system (Axiom Artis Zee, Siemens, Erlangen, Germany), see Figure 2A. For stenting of the ICA, the stenosis was first passed by a filter wire. In a second step, the self expandable stent was placed in the stenosis and postdilated with a PTA balloon, which was inflated for a short period (seconds, two minutes at maximum) to dilate the stenosis and the stent. Predilatation before stenting was not performed in this case series. The insufflation leads to flow arrest in the respective vessel, which did not need to be confirmed angiographically in our PTAs.

For BOT, a double lumen balloon occlusion catheter was placed in the cervical segment of the ICA. During the balloon inflation, flow arrest was confirmed by injection of contrast agent through the normal lumen of the balloon catheter resulting in flow stagnation distally to the balloon. Collateral flow was visualized by DSA and contrast injection into the dominant vertebral artery and the contralateral ICA using a diagnostic 5F catheter introduced transfemorally from the contralateral groin.

#### Near-Infrared Spectroscopy Recordings

A whole-head fiber holder of the FOIRE-3000 CW-NIRS system (Shimadzu, Japan) was mounted during preparation of the patients for endovascular intervention. The glass fibers of the optodes cause absorption of X-rays, whereas the plastic parts of the holder cap allow the radiation to pass, see Figure 2B. Although in the subtraction image (Figure 2C) the optodes in general do not reduce the visibility of cerebral blood vessels, we developed a standardized optode montage to provide the interventionalist with an unrestricted field of view of the main cerebral arteries, but still allows NIRS to cover parts of the vascular territories of the middle and anterior cerebral arteries (MCA and ACA) in both hemispheres.

To illustrate the vascular territories of the main cerebral arteries and their monitoring by CW-NIRS, we have exemplarily applied selective vessel-encoded arterial spin labeling (ASL) in a healthy subject. This technique enables noninvasive evaluation of vascular territories and collateral blood flow.^23^ Selective ASL was acquired on a Siemens 3T Verio MRI scanner (Siemens, Erlangen, Germany). Tagging duration was 1,375 ms for the tagging pulse train. The post labeling delay was 1,000 ms and recurrence and echo time were 3,000 ms and 52 ms, respectively. A total of 120 images were acquired for a set of 6 cycles. Analysis and computation of selective ASL maps were performed using Matlab programs (MathWorks, Natick, MA, USA).

**Figure 3:**
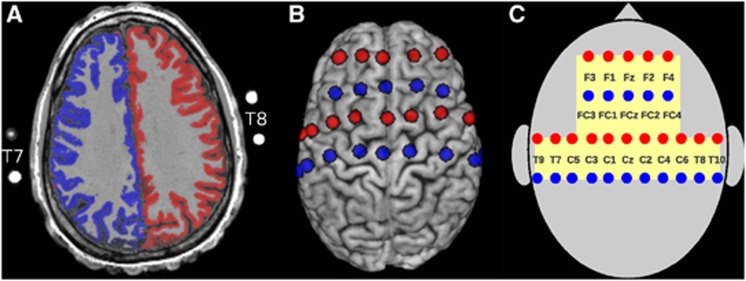
(**A**) Representation of the vascular territories supplied by the left (blue) and right internal carotid artery (ICA) (red) as assessed by selective arterial spin labeling (ASL) on the anatomy of a healthy subject. A cortex mask was applied to restrict the representation to cortical structures. Outside the skull magnetic resonance (MR)-visible markers (nitroglycerin medication capsules) of some near infrared spectroscopy (NIRS) optode positions are visible. (**B**) Projection of the optode positions (transmitters in red and receivers in blue) onto the skull-stripped brain of the same subject. Lateral views are available in Supplementary Figure S1 of the Supplementary Information. (**C**) Schematic representation of the optode montage with reference to approximate positions in the 10/10 electroencephalography system. Each optode pair spaced at 30 mm constitutes an NIRS channel. The yellow-shaded region is monitored by NIRS. This spatial layout is used in Figure 6 for representation of the results in all patients.

Figure 3A illustrates the vascular territories supplied by the left (blue) and right ICA (red) as an overlay onto an axial slice of the subject's structural MRI (overlays from selective labeling of the posterior circulation were excluded). Thirty-two magnetic resonance-visible surface markers (nitroglycerin medication capsules, four of them visible in the imaging slice on A) were placed inside the optode holders of the NIRS cap to indicate the positions of transmitters and receivers. One additional marker was used to identify the right hemisphere unequivocally. To estimate the optode positions over the brain, spheres centered at the marker positions were inflated computationally until they intersected with a binary brain mask of that subject. In Figure 3B, the approximate positions of transmitters (red) and receivers (blue) on the brain surface are shown. For later reference, we introduce in Figure 3C the spatial layout of the used optode montage. The yellow shaded area on the skull is monitored by NIRS. Channels are defined by the shortest distance (30 mm) between one transmitter and one receiver and indicated by approximate electroencephalography positions of the 10/10 system.

The FOIRE-3000 measures light attenuation at three wavelengths (780, 805, and 830 nm), which were converted to relative concentration changes in oxyhemoglobin Δ[HbO] and deoxyhemoglobin Δ[HbR] according to the modified Beer–Lambert law.^9^ From these, the changes in total hemoglobin Δ[HbT]=Δ[HbO]+Δ[HbR] and the difference Δ[HbD]=Δ[HbO]−Δ[HbR] were calculated. [HbT] is proportional to the cerebral blood volume (CBV) and [HbD] is a marker for blood oxygenation. The NIRS data were measured with 21 channels at 30 mm optode separation. Thirty-eight additional signals were recorded over longer distances but not included in the present analysis. Measurements covering the whole interventional sessions lasted for 44 to 167 minutes (87±47 min) and the sampling rate was 10 Hz. After montage of the NIRS optodes, the autocalibration routine of the FOIRE-3000 was used. According to its output all channels measuring over 30 mm optode distance showed sufficient signal quality in all patients. During the interventions, temporal markers were set manually in the FOIRE-3000 to indicate the time points when the angiographer injected contrast agent, inflated or deflated the occlusion balloon or performed any other intervention that might have had impact on cerebral hemodynamics. The specific target vessel was logged.

In endovascular interventions during wakefulness, head movements causing artifacts on CW-NIRS signals are frequent. This is especially true for patients handicapped by hypoxic damage of eloquent cortex or due to language and motor tasks required for neuropsychological testing during BOT. To minimize confusion by movement artifacts, we restricted our analysis of BOT data to the transition period from normal perfusion to vessel occlusion and to the first minutes of BOT. Patient S1 had a series of 20 occlusions of the left MCA lasting for 43±29 seconds, see Table 1.

### Data Processing

Movement artifacts were reduced in Δ[HbO] and Δ[HbR] signals using the MARA algorithm by Scholkmann *et al*^24^ in the implementation of the toolbox Homer2.^25^ Parameters were set for all patients equally. For artifact detection tMotion=0.5 second, tMask=2 seconds, STDEVthresh=6.0 and AMPthresh=0.02 was used. We manually excluded 2 seconds before and after occlusion start and termination from artifact removal to minimize the risk of erroneous ‘correction' of occlusion-associated signal changes. For spline interpolation during artifact removal *P*=0.1 was chosen to minimize over-fitting of artifacts.

**Figure 4:**
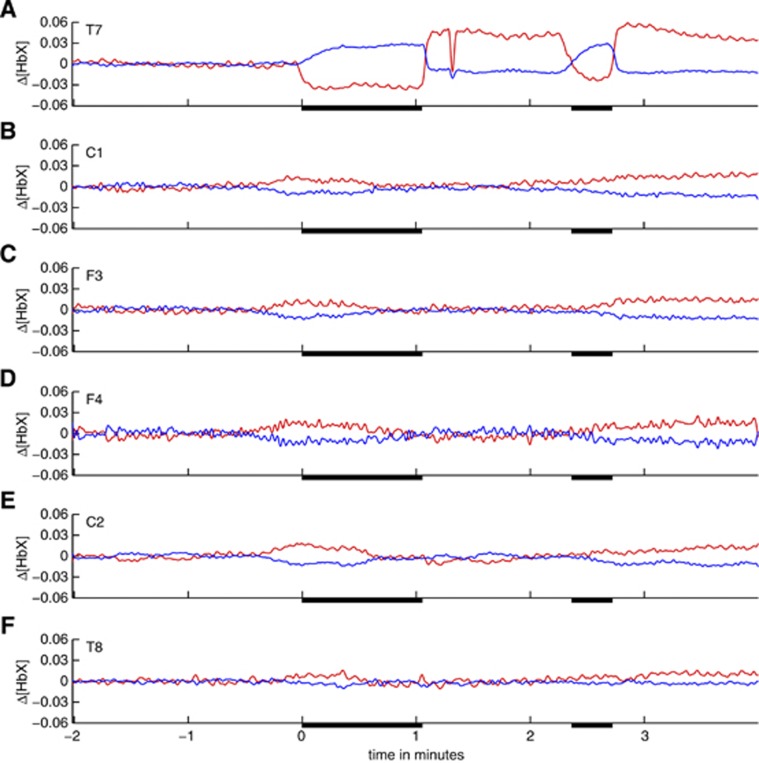
Temporal evolution of six selected near infrared spectroscopy (NIRS) signals in both hemispheres during two consecutive short-term balloon occlusions of the left middle cerebral artery (MCA) in patient S1. Signals were recorded at the following positions of the 10/10 electroencephalography system: (**A**) T7; (**B**) C1; (**C**) F3; (**D**) F4; (**E**) C2; and (**F**) T8. Relative hemoglobin concentration changes (red: Δ[HbO], blue: Δ[HbR], unit: mMolar*cm) are shown. All signals were low-pass filtered. Occlusion periods are indicated by black bars on the x-axes. Approximately 15 seconds after the first occlusion a bolus of contrast agent was injected into the left internal carotid artery (ICA). Opposite to the occlusions the blood dilution due to the bolus injection results in a concomitant short-term decrease in oxygenated and deoxygenated hemoglobin in the affected vascular territories (**A**).

**Figure 5:**
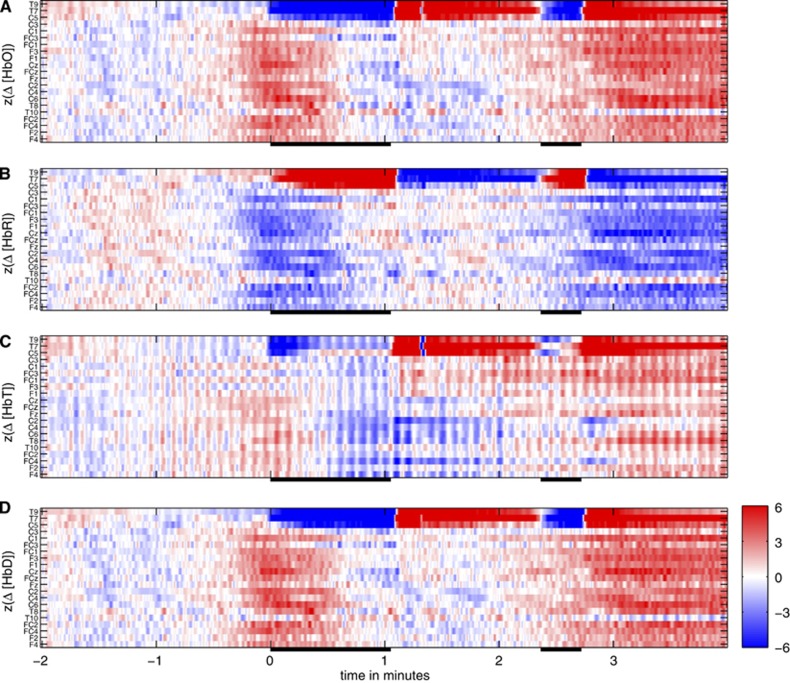
Temporal evolution of the *z*-scores of the near infrared spectroscopy (NIRS) signals with respect to the baseline of the last 120 seconds before temporary occlusion of the left middle cerebral artery (MCA) in patient S1. (**A**) Oxygenated hemoglobin. (**B**) Deoxygenated hemoglobin. (**C**) Total hemoglobin. (**D**) Hemoglobin difference. Similar to the usual electroencephalography display, each row represents one signal. Arrangement is from the left hemisphere over the midline to the right hemisphere (top to bottom) and within the hemispheres from central to frontal, see y-axes for exact positions in the 10/10 system.

After reduction in movement artifacts all NIRS signals were low-pass filtered using a digital implementation of a third order Butterworth filter with the 3 dB cutoff frequency at 0.4 Hz to remove heart rate and nonphysiologic high frequency components. The variations of the NIRS signals (Figure 4) were normalized to *z*-scores by subtraction of the mean and division by the standard deviation of the baseline during the last 2 minutes before vessel occlusion (Figure 5). Signals of NIRS are highly autocorrelated and in consequence subsequent samples are not statistically independent. To account for this, we used topographic maps of the mean *z*-scores in *t*_start_<*t*<*t*_end_ instead of the usual t-maps, which tend to yield spuriously high significance values in this setting. To avoid dependency on initial transient times and later movement artifacts, we chose *t*_start_=20 seconds and *t*_end_=60 seconds for occlusions lasting longer than 1  minute. Otherwise, the last 2/3 of vessel occlusion was analyzed.

To assess global spatial asymmetries of mean *z*-scores between both hemispheres in *t*_start_<*t*<*t*_end_ as would be expected when collaterals are insufficient, an exact permutation test^26^ was performed by iterating all possible allocations of homologous NIRS signal pairs (e.g., F3 versus F4) to one or the other hemisphere. The nonpaired locations on the midline (Fz, FCz, Cz) were excluded, leaving nine signal pairs for exhaustive redistribution. The difference z_occl_−z_contra_ was taken as test statistic. Asymmetries were interpreted as trends if *P*<0.05 and as significant if *P*<0.01.

All data processing and statistics was done with Matlab (MathWorks, version 7.0.4) scripts purpose-written by CR under the 64 bit version of Linux Ubuntu 12.04 LTS (Canonical Group Ltd., London, UK).

## Results

We present our results ordered according to the observed patterns of perfusion restriction as outlined in Figure 1.

### Patient S1: Repeated Middle Cerebral Artery Occlusion

As an example for occlusion of a vessel distal to the CoW without or with only minimal collaterals (e.g., via leptomeningeal collaterals), see Figure 1B, we present a coil embolization of a terminal ICA aneurysm with repeated short-term occlusions of the left proximal MCA in patient S1. This 35-year-old female presented with an incidentally detected terminal aneurysm of the left ICA. The angiographic documentation of the procedure is compiled in Supplementary Figure S2 of the Supplementary Information. To stabilize the aneurysm and to protect the origin of the MCA, a balloon occlusion technique (balloon remodeling) was applied ahead of and during the coil embolization of the aneurysm. Repeated temporary occlusion of the left distal ICA and the proximal part of the left MCA was performed (20 occlusions of 43±29 seconds within an intervention of >2 hours duration). Since the patient received general anesthesia, a simultaneous clinical neurologic examination during the transient MCA occlusions could not be performed. Permanent ischemic deficits were avoided by limiting the duration of each occlusion. The subsequent neurologic exam after the procedure and immediate postinterventional diffusion MRI were normal.

Figure 4 shows a period of 6 minutes of NIRS recording encompassing two subsequent MCA occlusions (#13 and 14). The NIRS signals recorded at scalp position T7 (in the core of the territory supplied by the MCA, panel A) are strongly modulated by the occlusions (black bars on the x-axes) that resulted in a stereotypical rapid and strong decrease in oxyhemoglobin and a slower and weaker increase in deoxyhemoglobin. [HbT] and [HbD] decrease during the MCA occlusions with a time scale of change comparable to oxyhemoglobin (data not shown). Deoxygenation in the affected vascular territory as indicated by decrease in [HbD] is consistent with the absence of collateral flow, as expected for an MCA occlusion, see Figure 1B. Near infrared spectroscopy signals recorded from other than left temporal channels exhibit a global low-amplitude counter-fluctuation of oxygenated and deoxygenated hemoglobin representing ultra-low frequency oscillations in the range of 0.001 Hz<*f*<0.01 Hz. As the data are not high-pass filtered and the intervention was performed under general anesthesia (minimal movement artifacts), similar oscillations are observable in large parts of the recording and apparently unrelated to the vessel occlusions. Color maps of the *z*-scores of Δ[HbO], Δ[HbR], Δ[HbT], and Δ[HbD] with respect to the last 120 seconds before the first shown MCA occlusion are displayed in Figure 5 for all NIRS signals. This pattern is very stereotypical for all MCA occlusions in patient S1 and emphasizes the spatially specific character of the occlusion-associated deoxygenation pattern (channels T9, T7, and C5 measuring from the core of the territory of the left MCA distal to the balloon occlusion) and the global character of the low frequency oscillations.

**Figure 6:**
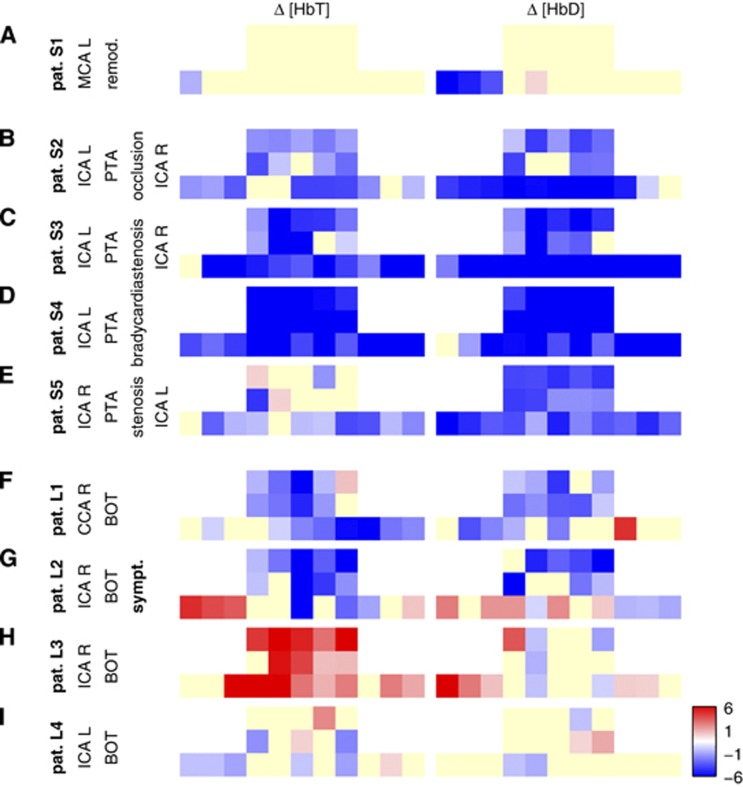
Spatial distribution of mean *z*-scores of Δ[HbT] and Δ[HbD] in 20 seconds<*t*<60 seconds (or the last 2/3 of shorter occlusions) for all patients. (**A**) Patient S1. (**B**) Patient S2. (**C**) Patient S3. (**D**) Patient S4. (**E**) Patient S5. (**F**) Patient L1. (**G**) Patient L2. (**H**) Patient L3. (**I**) Patient L4. The spatial layout is the same as introduced in Figure 3C. Channels where |*z*|<=1 are indicated in light yellow. For patient S1, the mean *z*-score over all 20 vessel occlusions is shown. BOT, balloon occlusion testing; CCA, common carotid artery; ICA, internal carotid artery; PTA, percutaneous transluminal angioplasty; MCA, middle cerebral artery.

The mean *z*-maps for Δ[HbT] and Δ[HbD] over *t*_start_<*t*<*t*_end_ and for all 20 MCA occlusions are shown in Figure 6A. For Δ[HbO] and Δ[HbR], the same is given in Supplementary Figure S23 of the Supplementary Information. At electroencephalography positions T9, T7, and C5 [HbD] decreases (*z*<−1), whereas except at T9 [HbT] (proportional to regional CBV) is rather stable (−1<*z*<0, not visible in the figure). The spatial asymmetry associated with the apparent higher deoxygenation ipsilateral to the MCA occlusions does not reach significance on the global level (*P*=0.20 for exact permutation test). The interhemispheric permutation test was designed to detect situations with insufficient collaterals via the CoW during ICA occlusion by revealing globally asymmetric changes between both hemispheres. With MCA occlusion, the core area of the MCA territory is prone to ischemia at the surface which is assessable by NIRS, excluding the watershed areas. These can be supplied by leptomeningeal collaterals arising from the ACA territory, which is left patent during MCA occlusion (Figure 1). In consequence, significantly asymmetric changes on global level could not be observed in patient S1. An analysis restricted to the core areas of the MCA territory (T9, T7, C5, C3, FC3, F3, and homologous contralateral positions) yielded trends toward interhemispheric differences for Δ[HbR] and Δ[HbD] (*P*=0.031 in both cases).

### Group 2: Short-Term Internal Carotid Artery Occlusions

Patients S2 to S5 underwent ICA stenting due to steno-occlusive carotid disease with diverse comorbidities, see Table 1. Short-term occlusions (duration 43±25 seconds, no significant difference to the 20 occlusions in patient S1, *P*=0.62 in a *t*-test) of the ICA were performed during PTA after stent placement. In patients S2, S3, and S5, no neurologic symptoms were present during or after the short-term occlusions. The intervention in patient S4 was performed under general anesthesia, thus neurologic deficits could not be assessed during the intervention. After the intervention, the neurologic exam was normal. Angiograms of patient S3 as well as detailed and temporally resolved NIRS data for this heterogenous patient group are compiled in the Supplementary Information. Here, we concentrate on the mean *z*-maps for Δ[HbT] and Δ[HbD] in the last 2/3 of the occlusions (Figures 6B to 6E).

A common feature of all patients with short-term occlusions of the ICA was a widespread decrease (*z*<−1) in oxygenation (measured by [HbD]) and CBV (proportional to [HbT]) without pronounced spatial asymmetry. This was most probably related to concomitant contralateral occlusive pathologies impeding effective collateral flow. Patients S2 and S3 had PTA in the left ICA, while the right ICA was either pseudo-occluded (S2, grading of 90% according to the North American Symptomatic Carotid Endarterectomy Trial, NASCET) or stenotic (S3, NASCET 54%). In patient S5 who received PTA in the right ICA and had an additional stenosis in the left ICA (NASCET 65%) the situation was opposite. The procedures resulted in bilaterally impaired carotid artery flow, leaving only the vertebral arteries as potential main collateral vessels. As a consequence, the deoxygenation during transient ICA occlusion was also visible in central and frontal regions not only over the ipsilateral, but also on the contralateral hemisphere. In all these patients, [HbD] decrease was explained by a concomitant [HbO] decrease and [HbR] increase (see Supplementary Figure S23).

Stenting in patient S4 was performed under general anesthesia. The patient developed bradycardia in response to PTA (decrease in heart rate from 45 to 33 b.p.m.) that triggered administration of atropine. Afterwards, hypertension was reported by the anesthesist. We interpret the observed global variation in oxygenation and CBV (see Figure 6D) as a consequence of decreased and reincreased supply of oxygenated blood due to bradycardia and subsequent hypertonia.

### Group 3: Prolonged Internal Carotid Artery Occlusions

Besides short-term occlusions that are mandatory either for vessel stabilization and protection (balloon remodeling) or during stent dilatation (PTA), in BOT the reason for selective arterial occlusion is to explicitly test the efficiency of collateral flow in the CoW, see Figure 1. During BOT, direction and extent of collateral flow is angiographically confirmed by two contrast injections into all brain supplying vessels at the beginning and later during the occlusion period. In addition, the duration of the occlusion needs to be long enough for possible (transient) neurologic deficits to become apparent. In the context of our study, this constitutes an advantage over the short-term occlusions, where collateral flow is not monitored and for patient protection occlusion times are kept too short for neurologic symptoms to become apparent. During BOT, three patients (L2 to L4) underwent ICA occlusion and L1 underwent common carotid artery occlusion (the ipsilateral external carotid artery was occluded by earlier surgery). Patient L2 developed transient neurologic symptoms indicating ischemia and BOT was aborted.

Patient L1 presented with an additional stenosis of the contralateral left ICA (NASCET 53%). We observed widespread CBV and oxygenation decrease (see Figure 6F as well as Supplementary Figures S15 and S16 of the Supplementary Information) during the first minute after vessel occlusion. The patient remained asymptomatic during the whole occlusion. In patient L2, the decrease in [HbT] and [HbD] was more pronounced over the affected right hemisphere and especially along the watershed areas between the right ACA and MCA territories (see channels F2, F4, and FC4 in Figure 6G; Supplementary Figures S17 and S18). This pattern remained stable for a longer period (Supplementary Figure S18) and CBV as measured by [HbT] increased in the contralateral temporal cortex after occlusion. A global increase in [HbT] and a smaller contralateral increase in [HbD] were detected in patient L3. In patient L4, [HbT] decreased moderately in the ipsilateral left temporal cortex. No consistent or spatially extended changes in oxygenation were observed.

Spatial asymmetry of the mean *z*-scores in 20 seconds<*t*<60 seconds was only significant for Δ[HbT] in patient L2, who developed neurologic symptoms during BOT (Figure 6G, *P*=0.0040). Interestingly, this asymmetry was accompanied by increased contralateral CBV. Without reaching significance on global level (*P*=0.27) also the oxygenation as measured by [HbD] was increased in the contralateral temporal cortex. In the asymptomatic patient L3, spatial asymmetry of Δ[HbT] was not significant (*P*=0.18).

## Discussion

In the present pilot study, we demonstrated the feasibility of multichannel CW-NIRS monitoring as a clinical application in patients receiving selective short-term (balloon remodeling and PTA) or prolonged occlusions (BOT) of brain supplying vessels during neuroangiological interventions. This combination of procedures allowed a validation of CW-NIRS signal changes by cerebral angiography (as a current gold standard). Multichannel CW-NIRS monitoring may provide additional information about altered hemodynamics as may occur during neurointervention and other potentially critical procedures. While the short-term occlusions are mandatory during certain endovascular interventions, the rationale for BOT is to probe maintenance of sufficient collateral flow along the arteries of the CoW during transient interruption of blood supply of one carotid artery without bearing the risk of acute ischemic complications.

With transient occlusion of blood vessels either distally or proximally to the CoW, two fundamentally different patterns were observed:

1. Pattern 1: Repeated occlusion of the MCA in patient S1 preserved the perfusion of the ACA and resulted in reduced perfusion exclusively in the territory of the MCA, irrespective of collateral flow via the CoW (Acom and Pcom)—in keeping with the site of occlusion distal to collaterals, see Figure 1B. Occlusion of MCA induced an immediate arrest of arterial inflow, whereas venous outflow was still open. The resulting decrease in CBV (Figure 5C, obscured in Figure 6A because −1<*z*<0 in the last 2/3 of the occlusions) is in keeping with the initially rapid decrease in [HbT] in the vascular territory of the MCA, followed by a prolonged decrease in [HbO] (Figure 5A) and a slower increase in [HbR] and [HbD] (Figures 5B and 5D). Oxygen is still withdrawn from the remaining stagnant blood due to the continuing metabolic demands of the tissue. The main effect was detected in the center of the MCA territory, with less pronounced effects at the borders—explained by leptomeningeal collateralization from the adjacent arteries along the watershed areas.
2. Pattern 2: An occlusion of the ICA may affect both the territories of the MCA and of the ACA, but allows for collateral circulation via the CoW depending on the supply by the Acom and Pcom:

(A) In patient L2, collateral circulation was insufficient (‘bad' collaterals, see Figure 1D for one but not the only possible underlying vascular anatomy) as indicated by the clinical deficits and confirmed by DSA. Correspondingly, severe and significantly asymmetric changes were observed for [HbT] in multiple NIRS channels that encompassed the territories of the MCA and ACA of both hemispheres (Figure 6G). The main decrease in [HbT] and [HbD] was observed along the watershed area between the ipsilateral right ACA and MCA, which represents a predilection area of hemodynamic infarctions due to carotid artery pathologies. Interestingly, [HbT] and [HbD] increased in the contralateral left temporal cortex, indicating the potential for compensatory collateral flow, which does not pass the CoW in this patient.

(B) In patients L1, L3, and L4, collateral circulation was sufficient to prevent neurologic symptoms (‘good' collaterals, see Figure 1C for a possible scenario) both clinically and by DSA. With the exception of patient L1, who had contralateral ICA stenosis, relative changes in all hemoglobin types were less pronounced and more symmetric, see Figures 6F, 6H, and 6I.

During the short-term occlusions in patients S2 to S5 we observed modulations in [HbT] and [HbD]. Due to concomitant contralateral occlusive pathology (patients S2, S3, and S5) or systemic effects (bradycardia and hypertension, patient S4) these changes were rather symmetric between hemispheres. These findings indicate that multichannel CW-NIRS is sensitive to hemodynamic effects resulting in insufficient collateral flow associated with temporary occlusion of cerebral arteries. Our observations in patients S1 and L2 indicate that these effects are *regionally specific* in the sense that occlusion-related signal variations depend on the position of the optodes over the vascular territories of the intracerebral arteries and the watershed regions.

Recently, it has been observed that CW-NIRS may produce results only poorly related or completely unrelated to neuronal activity, rather caused by systemic cardio-vascular or extracranial vascular changes.^27, 28, 29, 30, 31, 32, 33, 34, 35^ Several recommendations have been proposed to minimize the risk of misinterpretations. Systemic quantities as scalp blood flow, heart rate, and arterial blood pressure should be used as regressors for estimates of hemoglobin concentration changes in CW-NIRS studies. Oxy- and deoxyhemoglobin should be analyzed simultaneously instead of oxyhemoglobin alone. In addition, monitoring of pCO2 seems to be highly important because of its large-gain effect directly modifying cerebro-vascular autoregulation itself, resulting in flow alterations exceeding 50% readily achievable by voluntary (or involuntary) hyperventilation.^36^

In our setting, we can exclude the alternative explanation that the observed signal changes could be mostly due to superficial extracranial vessels, since the intervention selectively occluded brain supplying vessels (ICA and MCA) and the presence or absence of intracerebral collateral flow was controlled by simultaneous DSA. In consequence, our observations are also *brain specific*.

The novelty of our approach is to record Δ[HbO], Δ[HbR] and the derived quantities Δ[HbT], Δ[HbD] simultaneously with neurointervention in brain areas covering the cerebral cortex along widespread cerebro-vascular territories of the MCA and ACA. As time resolution of the used CW-NIRS system is in the subsecond range and optodes were placed at 30 mm spacing, our set-up is sensitive to fast and regional changes in collateral flow. So far, only a limited number of studies have reported NIRS monitoring during endovascular neuroradiologic interventions. Hernandez-Avila *et al*,^14^ Horie *et al*,^17^ Bhatia *et al*,^18^ and Mazzeo *et al.*^22^ employed different versions of the two-channel cerebral oxymeter INVOS (Somanetics, Troy, MI, USA) placed on the forehead and a sample interval of at least several seconds. Despite their limitations in spatial coverage and temporal resolution, all studies agreed that NIRS monitoring is of diagnostic value for early detection of cerebral oxygen desaturation during neuroangiographic intervention. The NIRS technology was specifically applied during BOT in refs. 16,19 and exemplary cases are shown in refs. 20,21. Calderon-Arnulphi *et al*^19^ applied a two-channel frequency-domain NIRS device that allowed absolute measurement of hemoglobin concentrations (Oxiplex TS, ISS Inc., Champaign, IL, USA). The authors concluded that an ipsilateral decrease in [HbO] discriminated ischemic events best (*P*<0.03). They observed a contralateral compensatory increase in oxygenation in a patient that developed neurologic symptoms under BOT. Our observations in patient L2 (Figure 6G; Supplementary Figure S23g) is in keeping with these studies, indicating widespread compensatory remote perfusion changes. Interestingly, the contralateral CBV increase was the only change in our patients that revealed a significantly asymmetric pattern (*P*=0.0040). Failing to reach significance a similar observation was made for the nonsymptomatic patient L3. We thus hypothesize that asymmetric compensatory perfusion and oxygenation increase as measured by CW-NIRS could be an early predictor of BOTs that indicate perfusion-related neurologic symptoms emerging at later time frames. Notably, a larger number of symptomatic patients are required to further support this hypothesis.

### Human Stroke Model

An important added value of CW-NIRS during neuroangiography is to enable real-time monitoring of cerebral oxygenation changes during vessel occlusions that resemble the spontaneous patho-physiologic conditions during transient ischemic attacks and stroke. With the exception of continuously monitored surgical procedures as cardiac or aortic surgery, where stroke risk is remarkably increased (see e.g. ref. 37 for review), the exact timing of stroke-related cerebral vessel occlusion is unpredictable and thus its onset can usually not be documented by imaging procedures in practice. The NIRS monitoring of routinely performed neurointerventional procedures as short-term vessel occlusion and BOT may provide valuable surrogate data regarding vascular changes that may occur in a similar manner during acute stroke. While changes in oxygenation can be assessed in real time with CW-NIRS, related clinical symptoms remain transient as occlusions can be stopped within seconds.

Selective vessel occlusion necessary during certain types of interventional procedures allows simulating two types of cerebro-vascular events:

1. Stroke with minor clinical deficits such as ICA occlusions that occur in patients with sufficient CoW collaterals;
2. Strokes with major clinical deficits such as ICA occlusions in patients without sufficient CoW collaterals or the majority of proximal MCA occlusions where CoW collaterals have no effect.

On the basis of magnetic resonance-angiographic findings, an entirely complete CoW was identified in 55% of 75 patients with ICA stenosis or occlusion who showed only mild symptoms. Remarkably, in a control group of 100 healthy subjects only 36% had an entirely complete CoW.^38^ This selection bias in the mildly symptomatic group emphasizes the importance of good collaterals in stroke and steno-occlusive disease.

Once CW-NIRS is validated with transitions to vessel occlusion and back to revascularization during mechanical thrombectomy, this noninvasive technique may gain importance as a clinical tool for real-time and bedside monitoring of thrombolytic effects during intravenous thrombolysis, which is not controlled by DSA. Here, a means to continuously monitor perfusion changes is of considerable clinical importance because current practice is to apply standard dosages. The lack of knowledge about individually tailored dosages poses an increased risk of cerebral hemorrhage, which is in the range of 5% to 6% of cases.^39, 40^

Beyond BOT and stroke, multichannel CW-NIRS monitoring may add valuable information about circulation changes during peri-procedural monitoring as, e.g., treatment of vasospasm, cardiac or aortic surgery and remodeling techniques during endovascular aneurysm treatment. The latter was exemplarily demonstrated in patient S1 of the present report.

### Limitations

The data of this continuous study are currently limited to our first observations and report the feasibility of the suggested multimodal approach to investigate collateral flow in a realistic clinical setting. Our results are heterogenous and the number of BOT patients (four) was small. Other tertiary care centers that investigated 25 combined DSA-NIRS procedures over a 22-month sampling period reported a similar number of 5 BOTs in their cohort, from which one failed the test clinically.^19^ We aim to follow patients with BOT in a systematic way and increase the number of patients during the next years. The aim is to evaluate sensitivity and specificity of early asymmetry in hemoglobin changes as observed in our patient L2 for prediction of negative BOT outcome.

The CW-NIRS technology is limited to observation of concentration *changes* occurring during relatively short periods of time, integrates measurement of superficial with deeper layers and is relatively prone to movement artifacts. While other techniques like frequency-domain and time-domain NIRS would enable better separation of the signal origin as well as absolute concentration measurements, these technologies are still much more expensive, less mobile and have smaller number of channels.^12^

### Summary

We demonstrated the feasibility of simultaneous CW-NIRS and angiography of the intracranial vessels. In selected cases, multichannel CW-NIRS detected regionally and brain-specific hemodynamic changes corresponding to collateral flow during temporary occlusion of intracerebral arteries as proven by angiography.

## Ethics Statement

This pilot study was approved by the Kantonale Ethikkommision (KEK) Bern. Experiments were performed according to this institution's statement dated 4 May 2012, according to Swiss law and institutional guidelines.

## Figures and Tables

**Table 1:** Demographics and etiology as well as background information on performed interventions and NIRS measurements

*ID*	*Age (years)*	*Gender*	*Etiology*	*Intervention*	*NIRS measurement duration (minutes)*	*Site of occlusion*	*Number of occlusions*	*Occlusion duration (seconds)*	*Anesthesia*	*Remarks*
S1	35	F	Aneurysm in proximal left ICA	Balloon remodeling	143	Left MCA	20	43±29 (range 11–105)	General	—
S2	80	F	Steno-occlusive disease	PTA	44	Left ICA	1	20	Local	Restenosis of left ICA, pseudo-occlusion of right ICA (NASCET 90%)
S3	59	M	Steno-occlusive disease	PTA	70	Left ICA	1	31	Local	Stenosis in left (NASCET 57%) and right ICA (NASCET 54%)
S4	81	M	Steno-occlusive disease	PTA	167	Left ICA	1	45	General	Pseudo-occlusion of left ICA (NASCET 90%), bradycardia during PTA
S5	79	M	Steno-occlusive disease	PTA	75	Right ICA	1	77	Local	Stenosis in right (NASCET 70%) and left ICA (NASCET 65%)
L1	69	M	Laryngeal carcinoma right	BOT	38	Right CCA (no right ECA)	1	1,275	Local	Nonsymptomatic, stenosis of the left ICA (NASCET 53%)
L2	48	F	Giant aneurysm in right ICA	BOT	52	Right ICA	1	626	Local	Symptomatic
L3	25	F	Neurofibroma at skull base	BOT	65	Right ICA	1	2,060	Local	Nonsymptomatic
L4	59	M	Laryngeal carcinoma left	BOT	128	Left ICA	1	2,100	Local	Nonsymptomatic

BOT, balloon occlusion testing; CCA, common carotid artery; ICA, internal carotid artery; ECA, external carotid artery; PTA, percutaneous transluminal angioplasty; MCA, middle cerebral artery; NASCET, North American Symptomatic Carotid Endarterectomy Trial; NIRS, near infrared spectroscopy.
