# Supplementary material for: Monitoring cerebral oxygenation during balloon occlusion with multichannel NIRS
Source: J Cereb Blood Flow Metab. 2013 Dec 4;34(2):347–56. doi: 10.1038/jcbfm.2013.207 (PMC3915216; doi:10.1038/jcbfm.2013.207)
Supplement: Supplementary Information [file jcbfm2013207x1.pdf]

Monitoring Cerebral Oxygenation During Balloon Occlusion with Multi-Channel NIRS – Supplementary Information

Christian Rummel[*,a,1], Christoph Zubler[a], Gerhard Schroth[a], Jan Gralla[a], Kety Hsieh[a], Eugenio Abela[a,b], Martinus Hauf[a], Niklaus Meier[b], Rajeev K. Verma[a], Robert H. Andres[c], Arto Nirkko[b], Roland Wiest[a]

[a] *University Institute of Diagnostic and Interventional Neuroradiology, Inselspital, Bern University Hospital, University of Bern, Switzerland*

[b] *Department of Neurology, Inselspital, Bern University Hospital and University of Bern, Switzerland*

[c] *Department of Neurosurgery, Inselspital, Bern University Hospital, University of Bern, Switzerland*

---

[*] Corresponding author

[1] Christian Rummel, phone 0041 31 6328038, fax 0041 31 6324872, crummel@web.de

[2] CR and CZ contributed equally to this work. AN and RW share senior authorship.

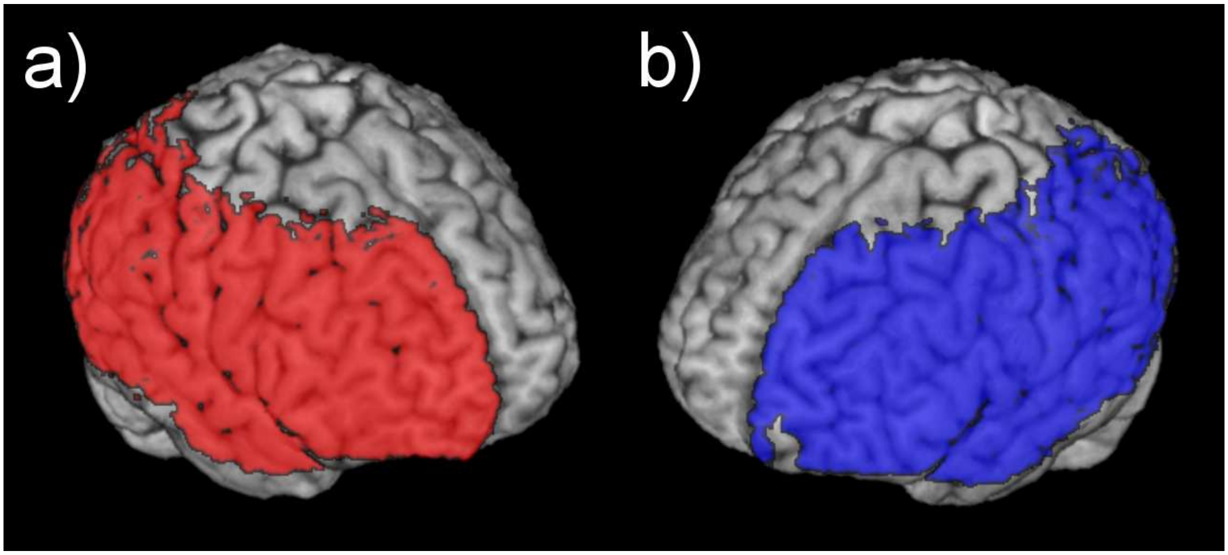

Figure S1: Display of selective arterial spin labeling (sASL) data on the structural magnetic resonance image (MRI) of a **healthy subject**, see main text. Note that due to the limitation to a stack of 120 slices the supply of the vertex is not represented. Shown is the vascular territory supplied by the right (panel a, red) and left (panel b, blue) internal carotid artery (ICA).

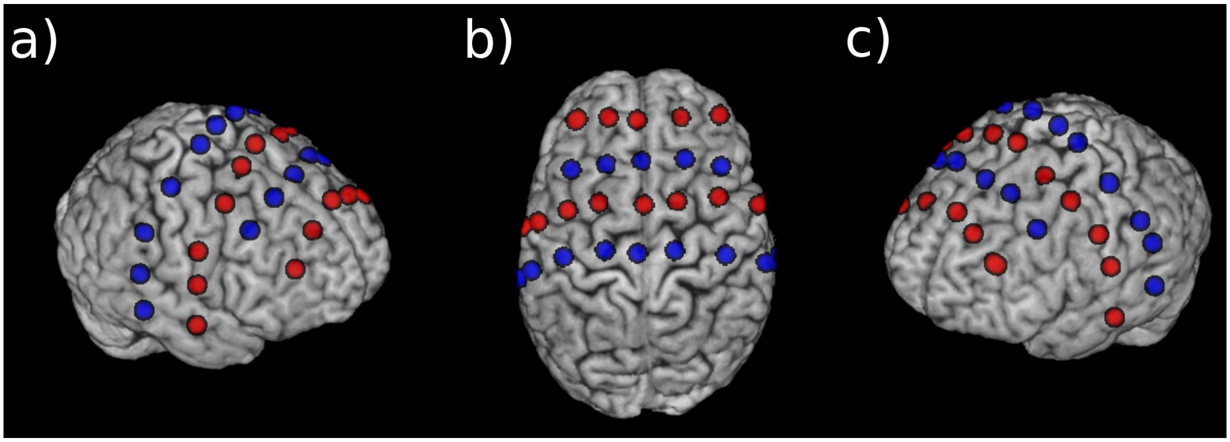

Figure S2: Approximate projection of optode positions onto the structural MRI of a **healthy subject**. Near infrared light transmitters are shown in red and receivers in blue. The MRI was acquired with nitroglycerine medication capsules as markers (high contrast) positioned inside the optode holders of the near infrared spectroscopy (NIRS) cap, see Fig. 3a of the main text. Coordinates of marker centers were identified manually and a brain mask was generated using the freely available software package FSL (`http://www.fmrib.ox.ac.uk/fsl/`). Using a self written Matlab script (MathWorks, Natick, MA, USA, version 7.0.4) the markers were inflated spherically until they intersected with the brain mask. The intersection points were used as optode locations on the brain. Approximate NIRS measurement points are centered between transmitter-receiver pairs. Panel b of the figure is identical to Fig. 3b of the main text.

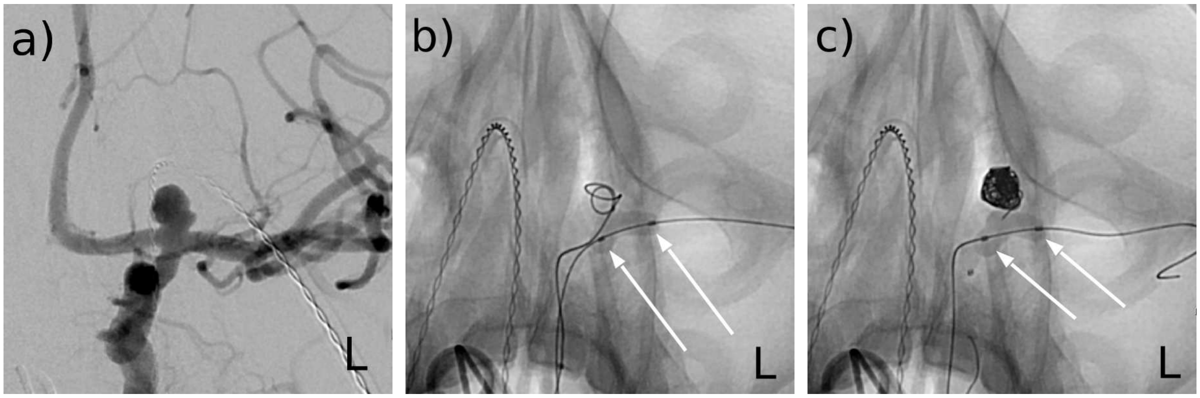

Figure S3: **Patient S1:** Coiling of a terminal aneurysm in the left ICA. Angiograms are shown a) before coiling, b) initial phase of endovascular treatment, c) after coiling. In panels b and c the remodeling balloon in the left proximal middle cerebral artery (MCA) is visible. Arrows point to the proximal and distal balloon markers.

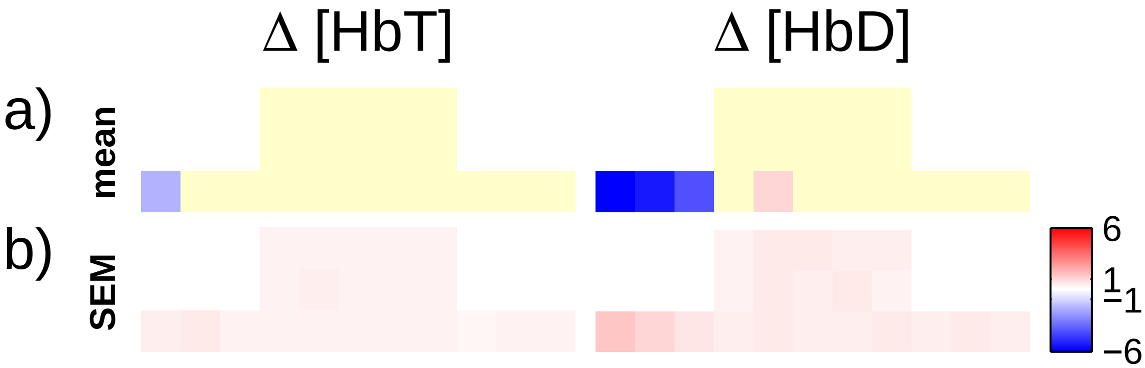

Figure S4: **Patient S1:** Regional z-score statistics during the last 2/3 of 20 short term occlusions of the left MCA. a) mean (also shown in Fig. 6a of the main text), b) standard error of the mean. Channel positions are defined in Fig. 3c of the main text. Regions with values $-1 < \langle z \rangle < 1$ are depicted in yellow.

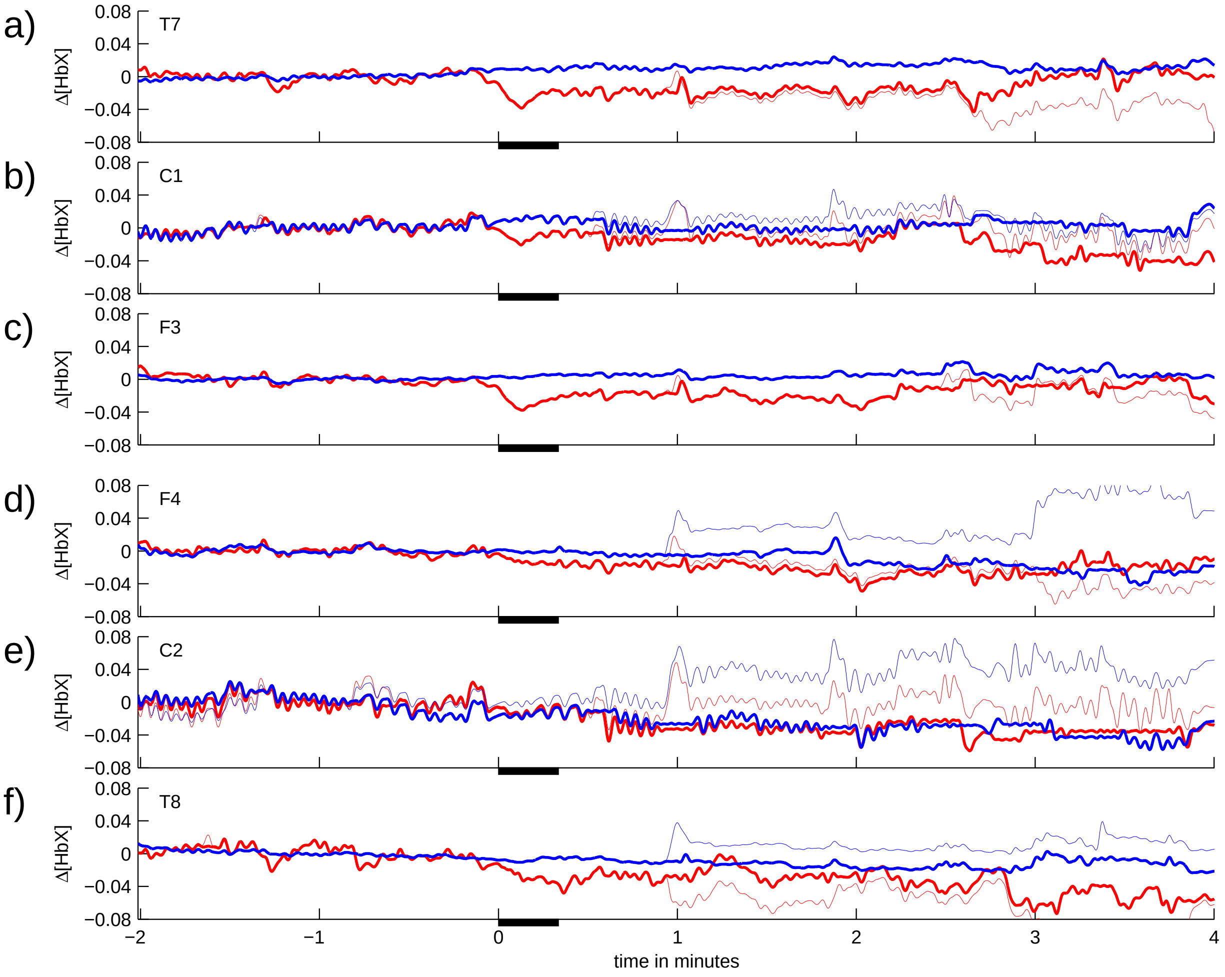

Figure S5: **Patient S2:** Time series of NIRS signals for selected optode locations on both hemispheres. The unit of concentration changes is mMolar·cm. Oxyhemoglobin is shown in red and deoxyhemoglobin in blue. Raw data is drawn as thin lines and movement artifact corrected data as thick lines. The figure arrangement is identical to Fig. 4 of the main text. Short-term occlusion of the left ICA during percutaneous transluminal angioplasy (PTA) is indicated by black bars on the x-axes. Although movement artifacts are often reduced, not all are satisfactorily removed by the movement artifact correction algorithm with parameters chosen identical for all patients, see main text.

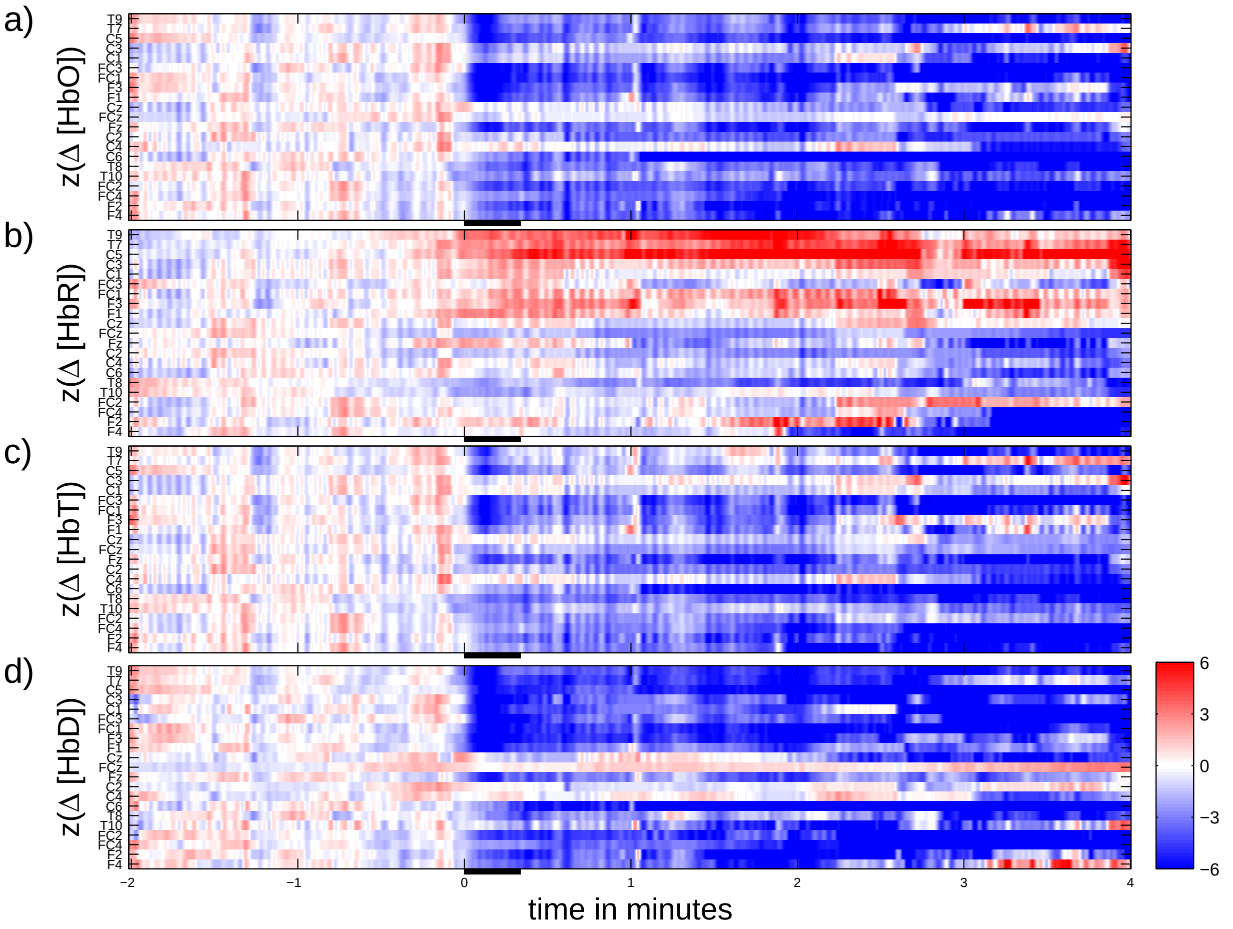

Figure S6: **Patient S2:** Color coded representation of z-scores of all movement artifact corrected NIRS signals with respect to the last 120 seconds before short-term occlusion of the left ICA (black bars on x-axes). The figure arrangement is identical to Fig. 5 of the main text. This patient has an additional contralateral pseudo-occlusion of the right ICA (NASCET 90%). As under these conditions only the vertebral arteries (VA) remain as collaterals during occlusion, this may explain the largely spatially symmetric reaction of NIRS signals. Note, however, that the occlusion initiated increase in deoxgenated hemoglobin is more pronounced in the left temporo-central cortex.

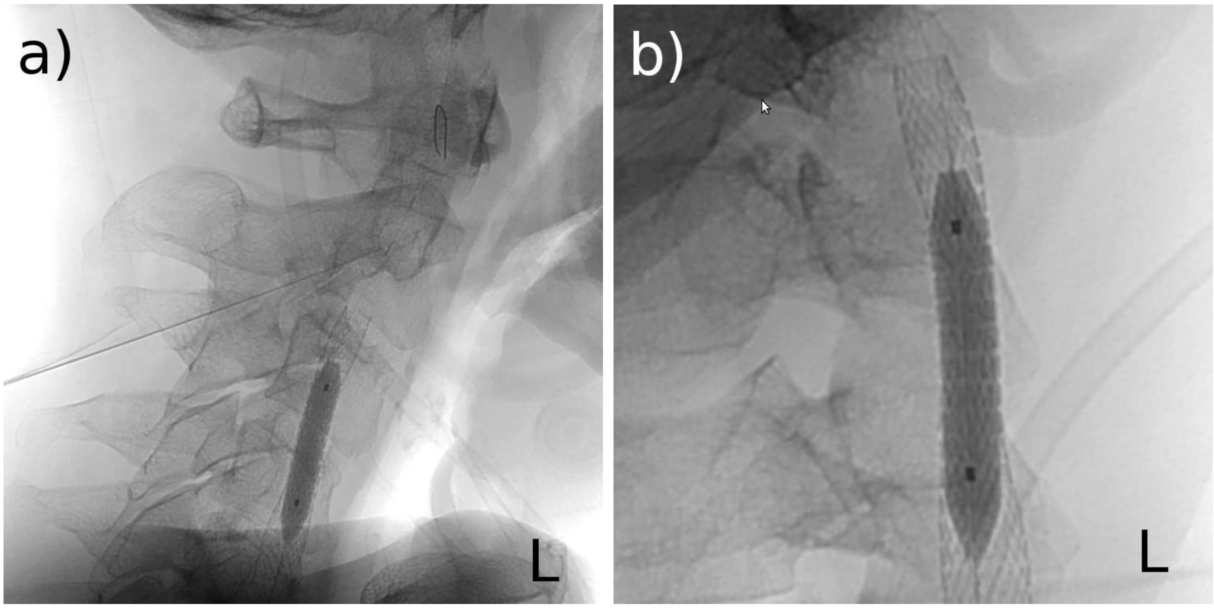

Figure S7: **Patient S3:** Angiogram during PTA after stent placement in the left ICA. The stent and the dilatation balloon are clearly visible.

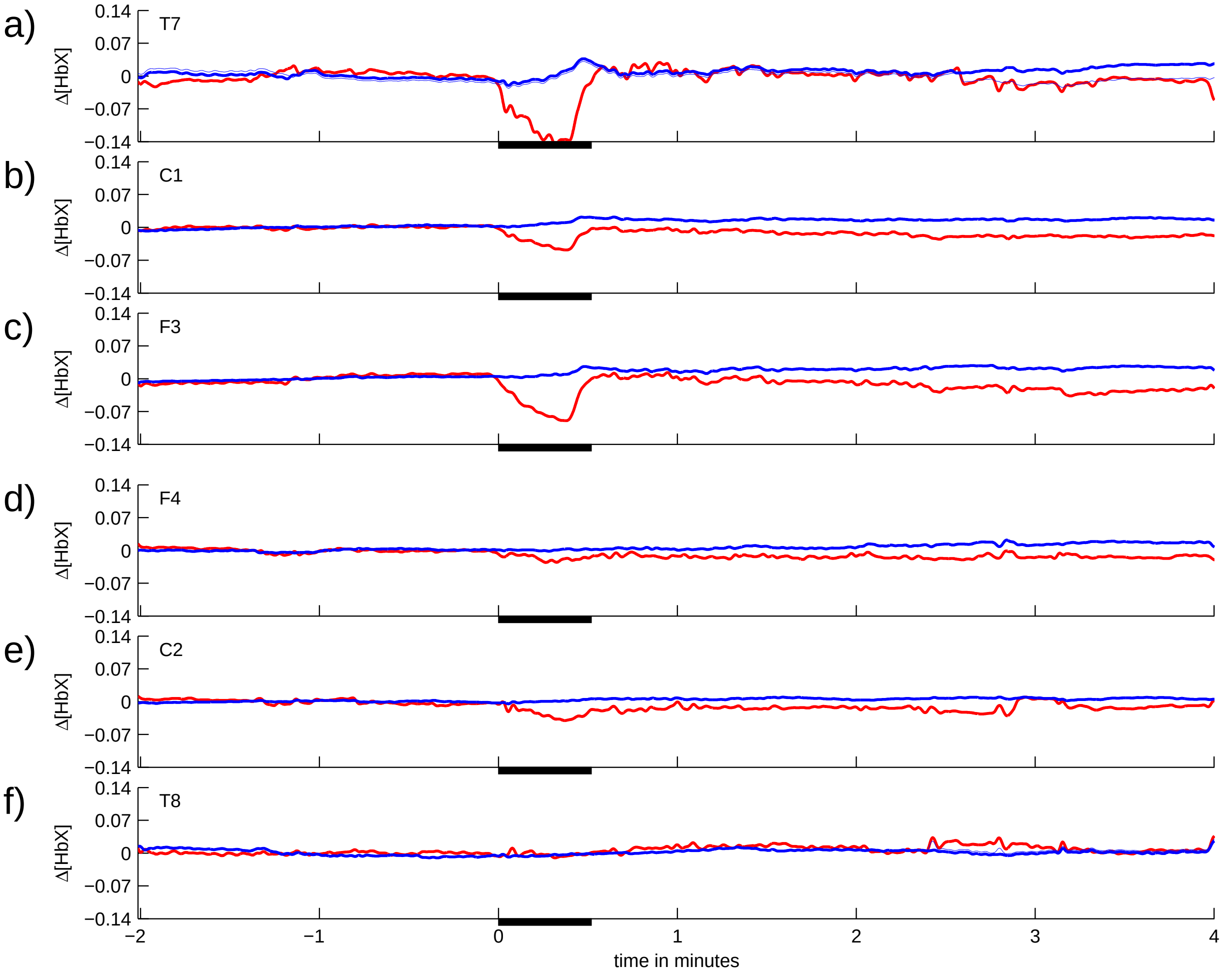

Figure S8: **Patient S3:** Selected NIRS time series during short-term occlusion of the left ICA (black bars on x-axes). The figure arrangement is as in Fig. S5.

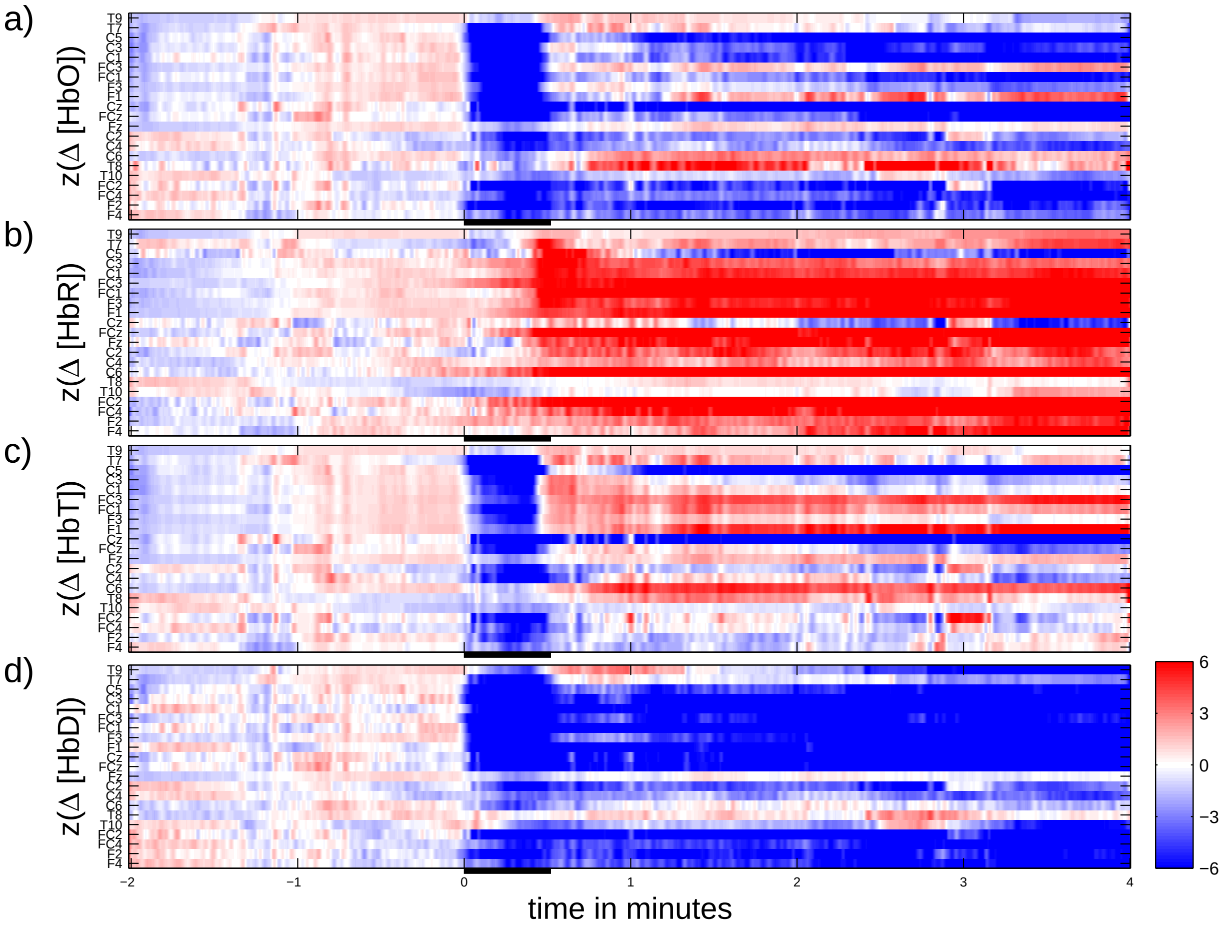

Figure S9: **Patient S3:** z-scores of all movement artifact corrected NIRS signals with respect to the last 120 seconds before short-term occlusion of the left ICA (black bars on x-axes). The figure arrangement is identical to Fig. S6. This patient also has a contralateral stenosis of the right ICA (NASCET 54%), which may explain the co-reaction of NIRS signals on the right hemisphere to the occlusion.

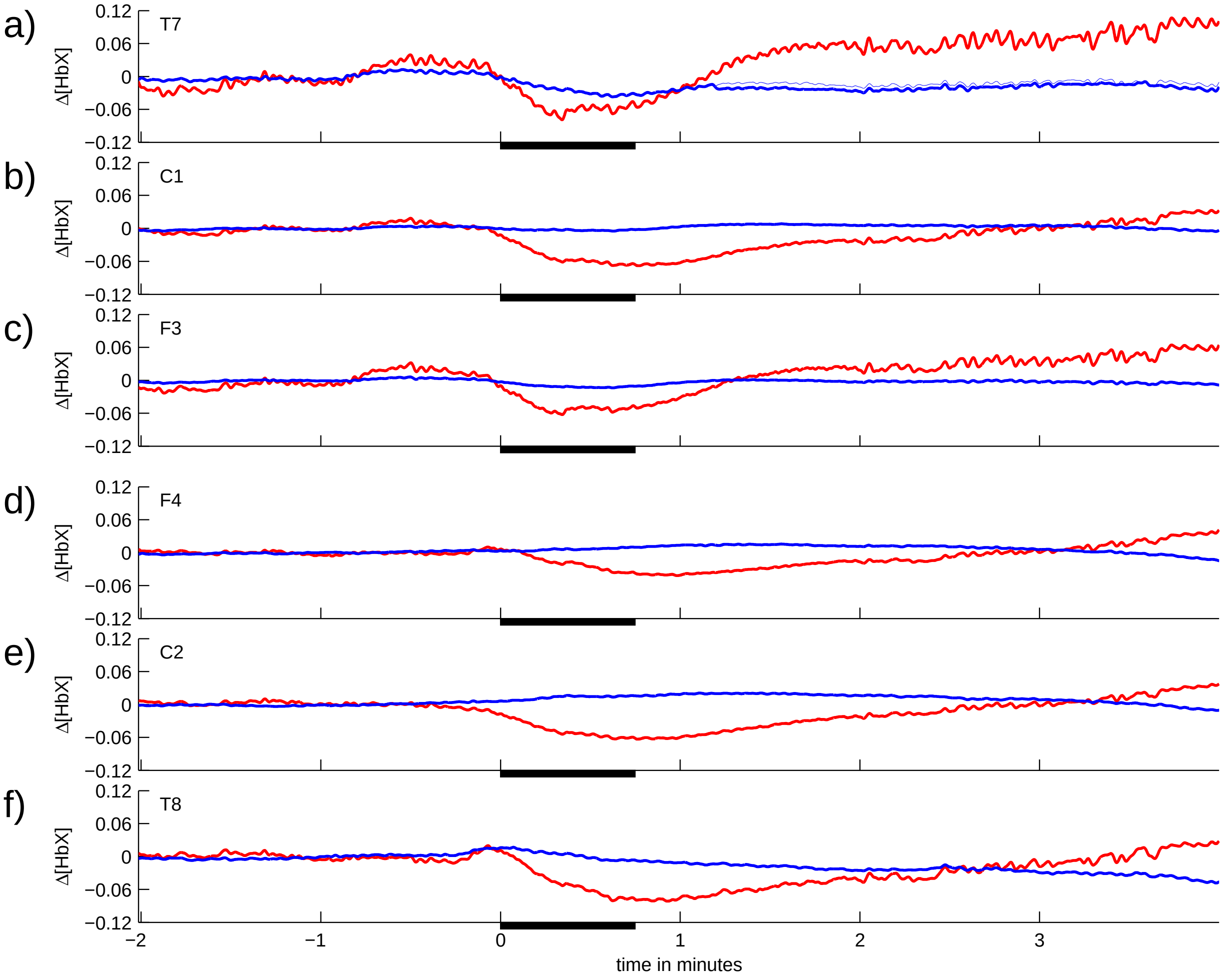

Figure S10: **Patient S4:** Selected NIRS time series during short-term occlusion of the left ICA (black bars on x-axes). The figure arrangement is as in Fig. S5.

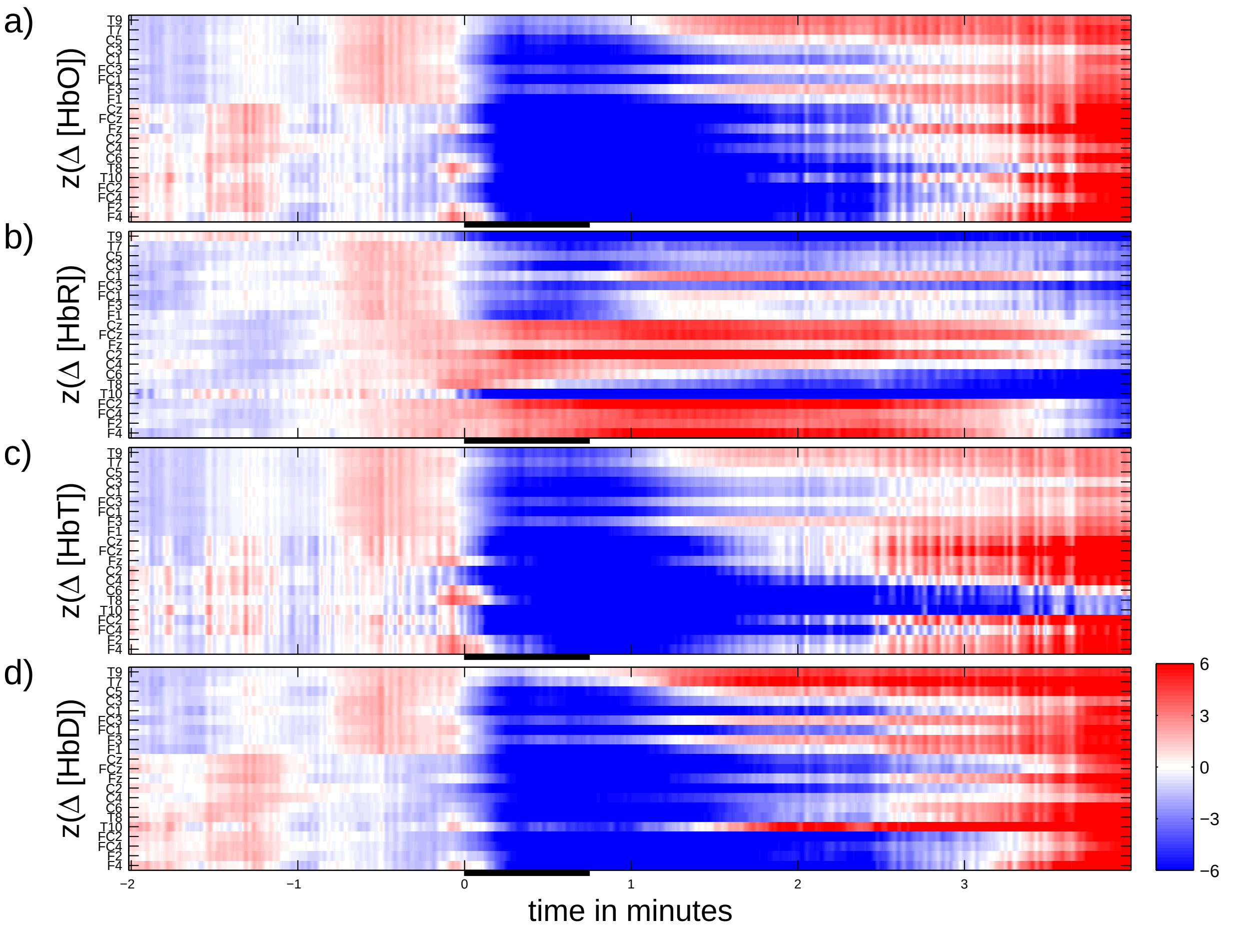

Figure S11: **Patient S4:** z-scores of all movement artifact corrected NIRS signals with respect to the last 120 seconds before short-term occlusion of the left ICA (black bars on x-axes). The figure arrangement is identical to Fig. S6. This intervention was performed under general anesthesia. The anesthesist reported a drop of heart rate from 45 to 33 bpm immediately after PTA start, which was treated by prompt Atropine administration. Subsequently, the anesthesist reported massive hypertonia. Bradycardia and hypertonia are systemic influences, which may explain the largely symmetric oxygenation drop and re-increase.

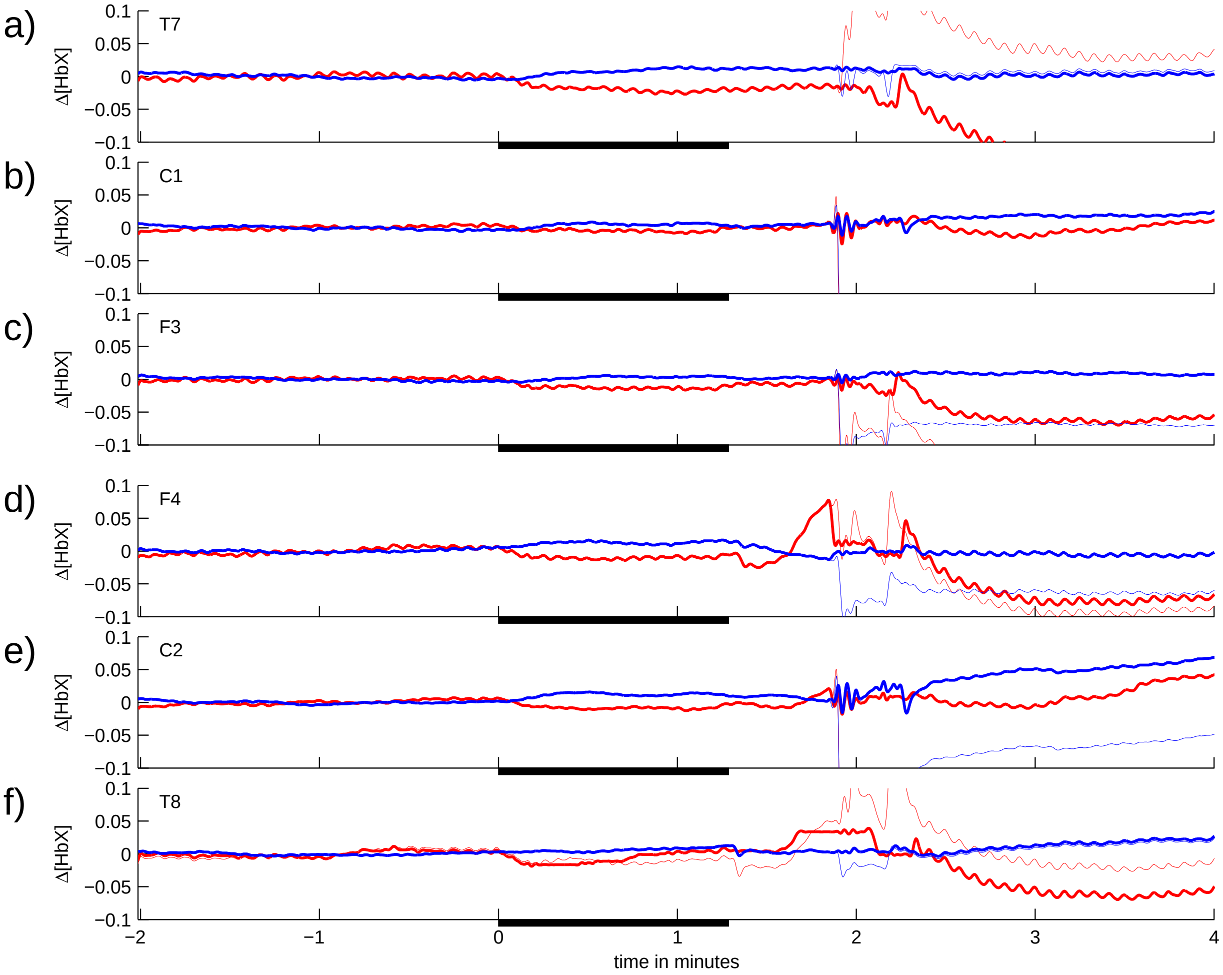

Figure S12: **Patient S5:** Selected NIRS time series during short-term occlusion of the right ICA (black bars on x-axes). The figure arrangement is as in Fig. S5. After termination of the occlusion a massive movement artifact corrupted the NIRS signals ($t \approx 2$min), which can only partly be reduced by the movement artifact reduction algorithm.

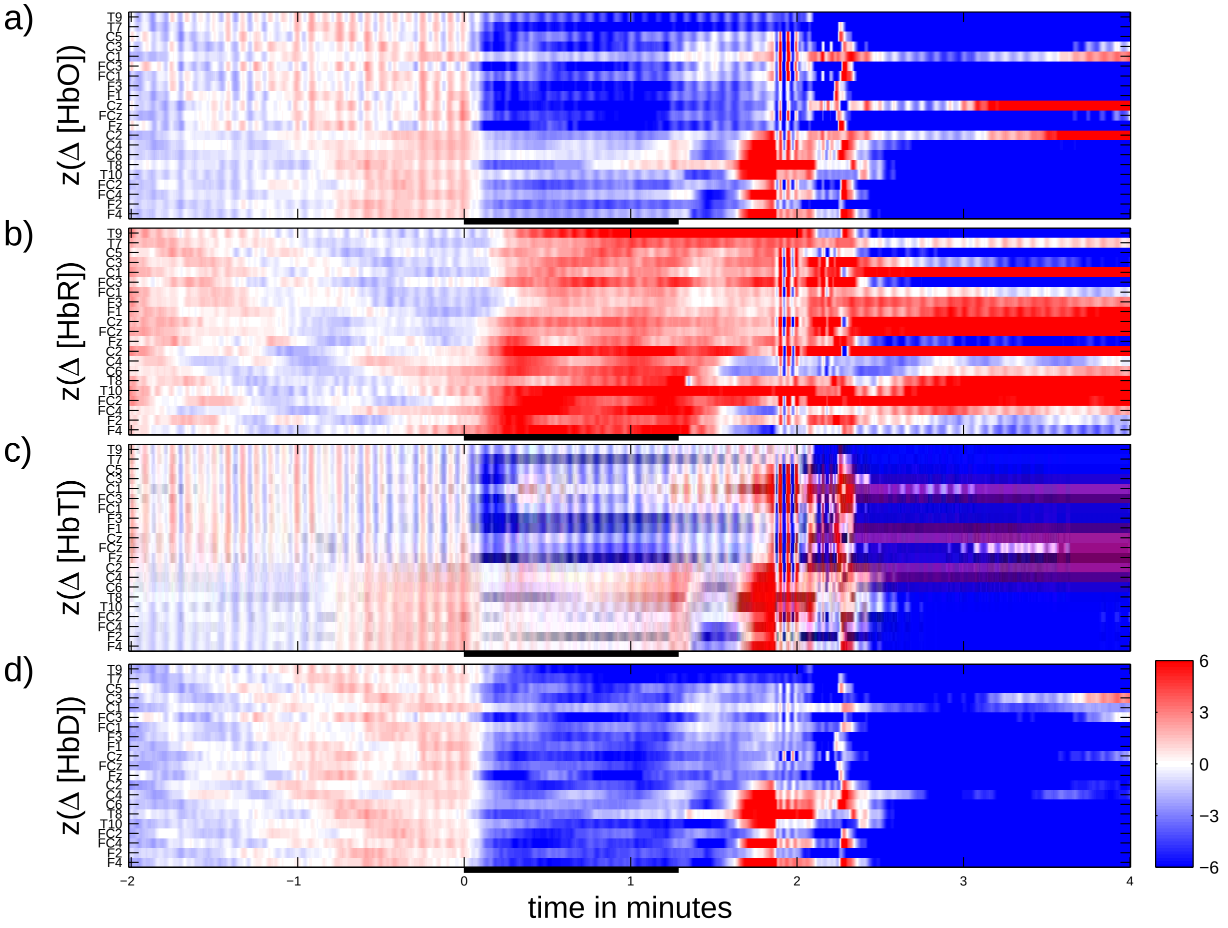

Figure S13: **Patient S5:** z-scores of all movement artifact corrected NIRS signals with respect to the last 120 seconds before short-term occlusion of the right ICA (black bars on x-axes). The figure arrangement is identical to Fig. S6. This patient also has a contralateral stenosis of the left ICA (NASCET 65%), which may explain the largely spatially symmetric reaction of NIRS signals to the occlusion.

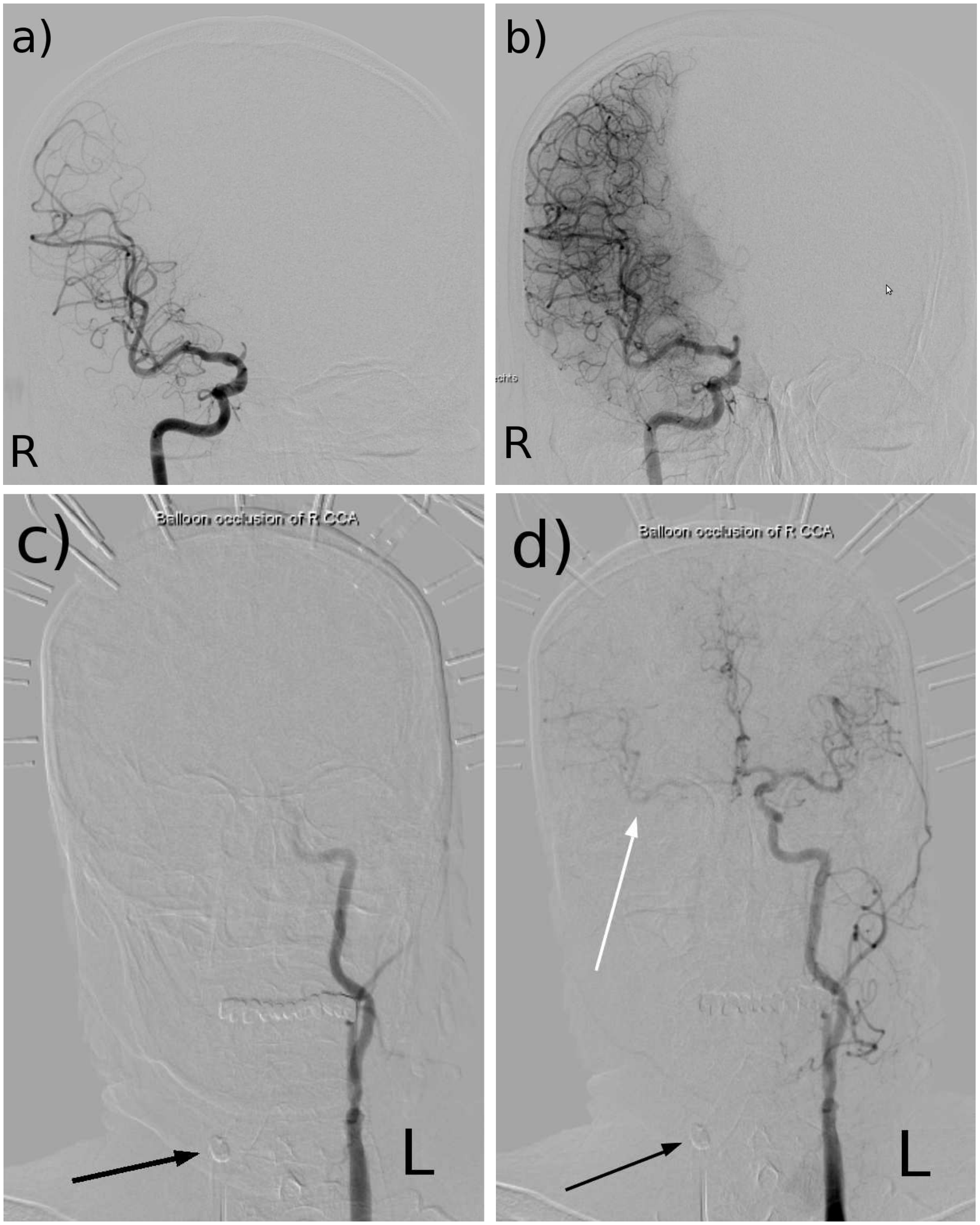

Figure S14: **Patient L1:** Digital subtraction angiography (DSA) before and during balloon occlusion of the right common carotid artery (CCA). a,b) Consecutive phases of contrast injection in the right CCA *before* occlusion. Shown is the vascular territory supplied by the right ICA. c,d) Consecutive phases of contrast injection in the left ICA *during* occlusion of the right CCA. The collateral flow via the Circle of Willis (CoW) towards the right hemisphere is visible in panel d (white arrow). The black arrows point to the occlusion balloon.

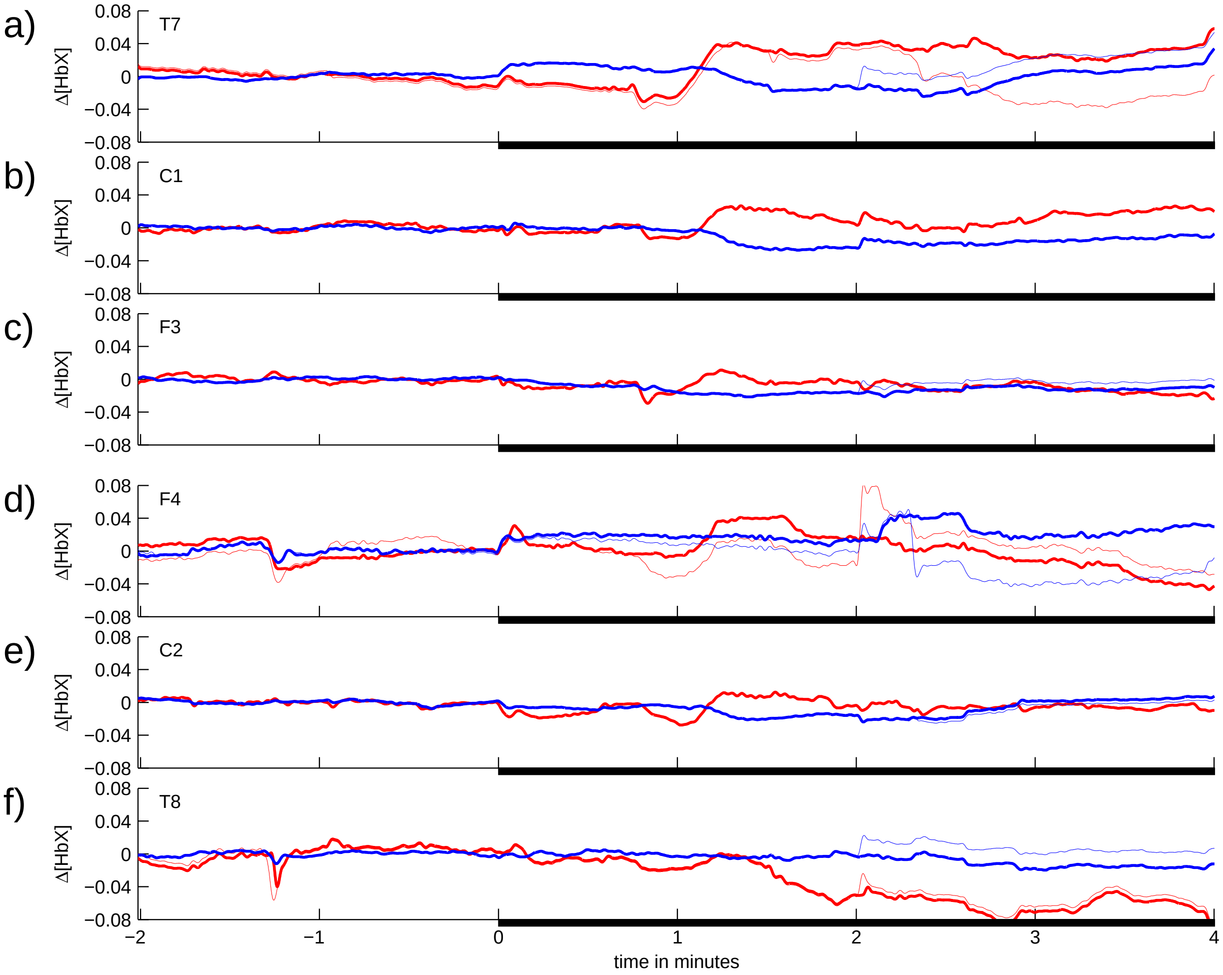

Figure S15: **Patient L1:** Selected NIRS time series during the first minutes of prolonged occlusion of the right CCA (black bars on x-axes). The figure arrangement is as in Fig. S5. A movement artifact that cannot be fully compensated by the movement artifact removal algorithm on all channels is visible on the NIRS signals approximately 2 minutes after occlusion start.

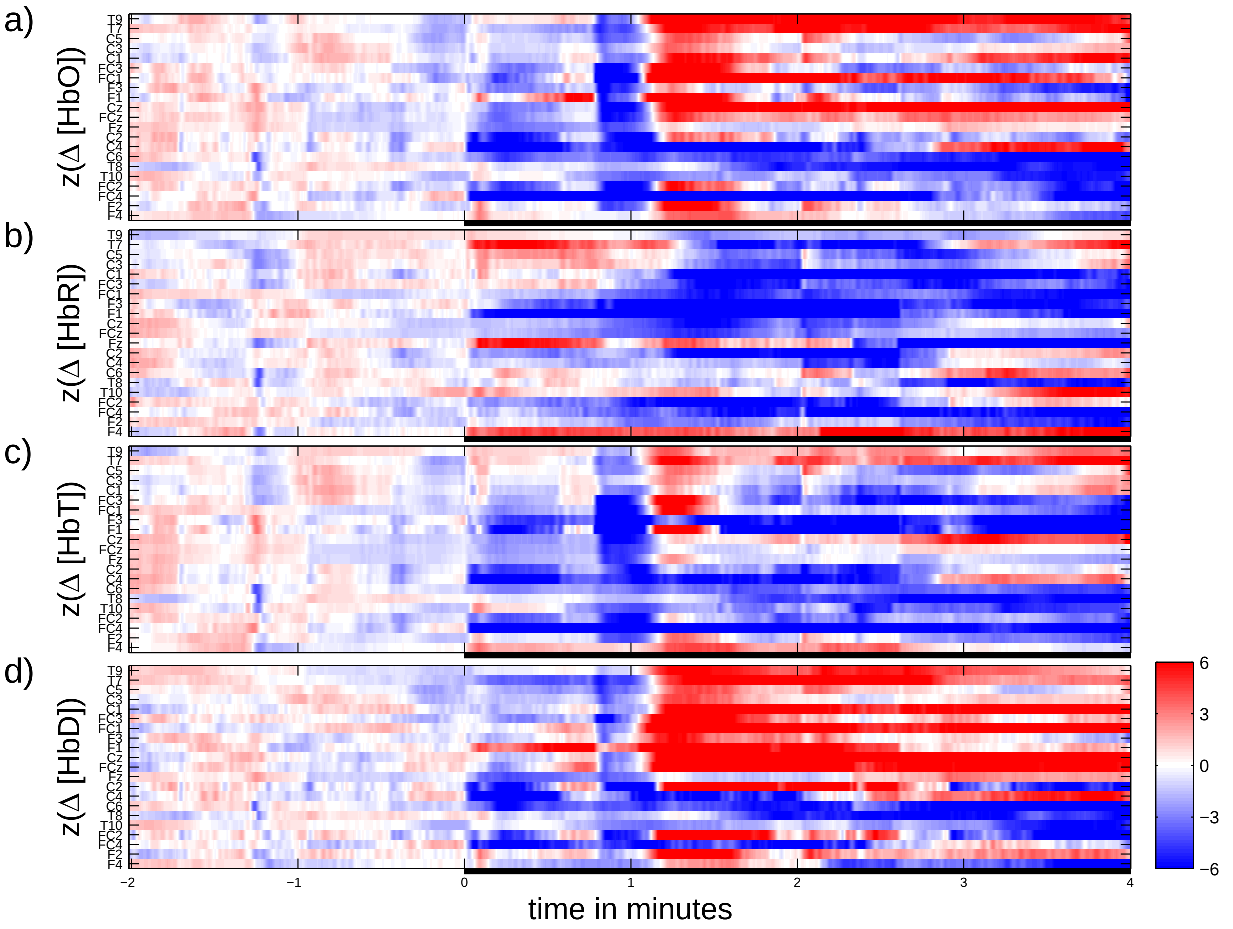

Figure S16: **Patient L1:** z-scores of all movement artifact corrected NIRS signals with respect to the last 120 seconds before prolonged occlusion of the right CCA (black bars on x-axes). The figure arrangement is identical to Fig. S6. In this patient the right ECA has been occluded by earlier surgery and there are additional contralateral stenoses in the left ICA (NASCET 53%). This situation may explain the co-reaction of NIRS signals measured from the left hemisphere immediately after occlusion start. Repeated neuropsychological testing showed no symptoms in this patient and contrast bolus injection revealed sufficient collateralization via the CoW, see Fig. S14d.

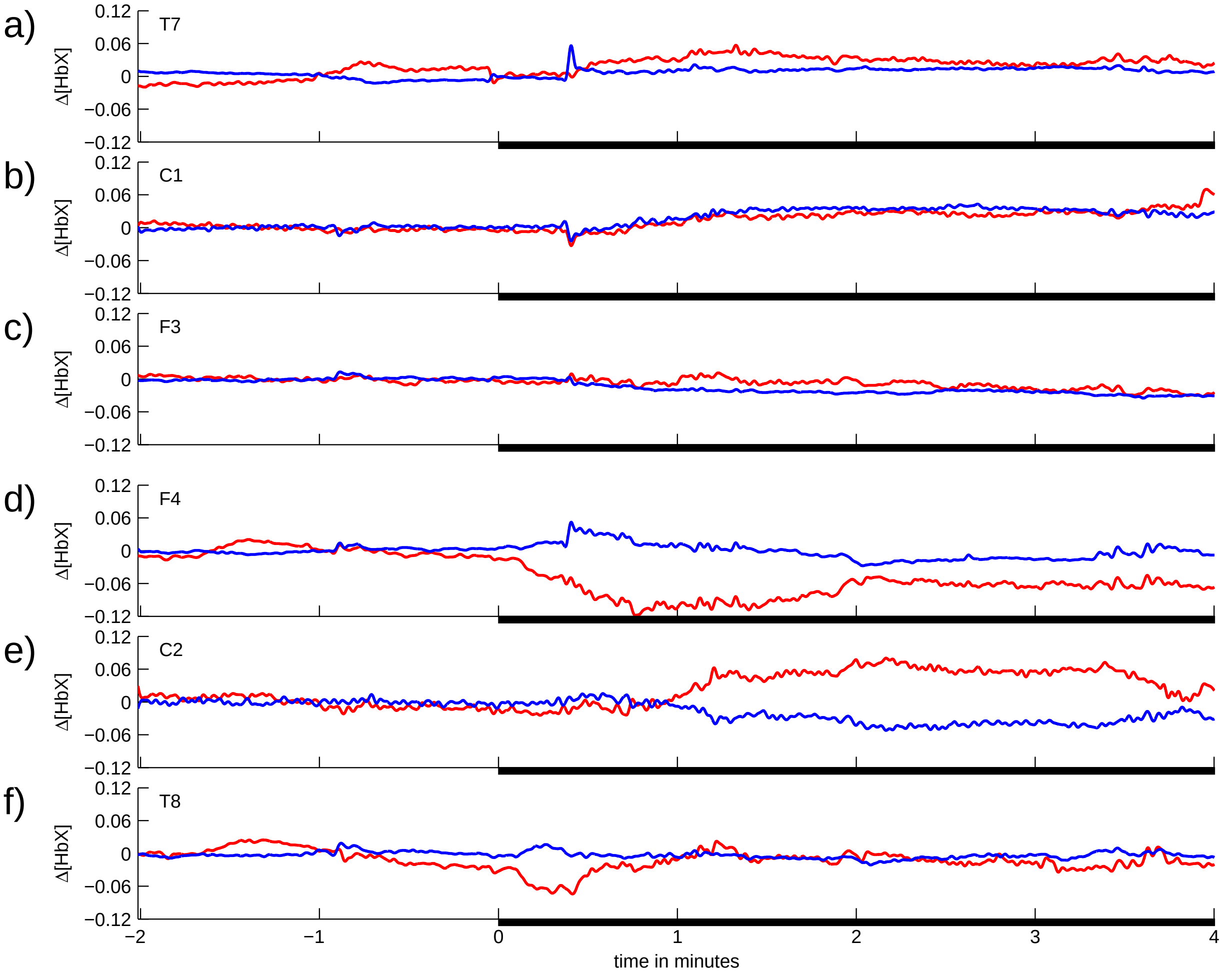

Figure S17: **Patient L2:** Selected NIRS time series during the first minutes of prolonged occlusion of the right ICA (black bars on x-axes). The figure arrangement is as in Fig. S5. An uncompensated movement artifact is visible on the NIRS signals approximately 25 seconds after occlusion start.

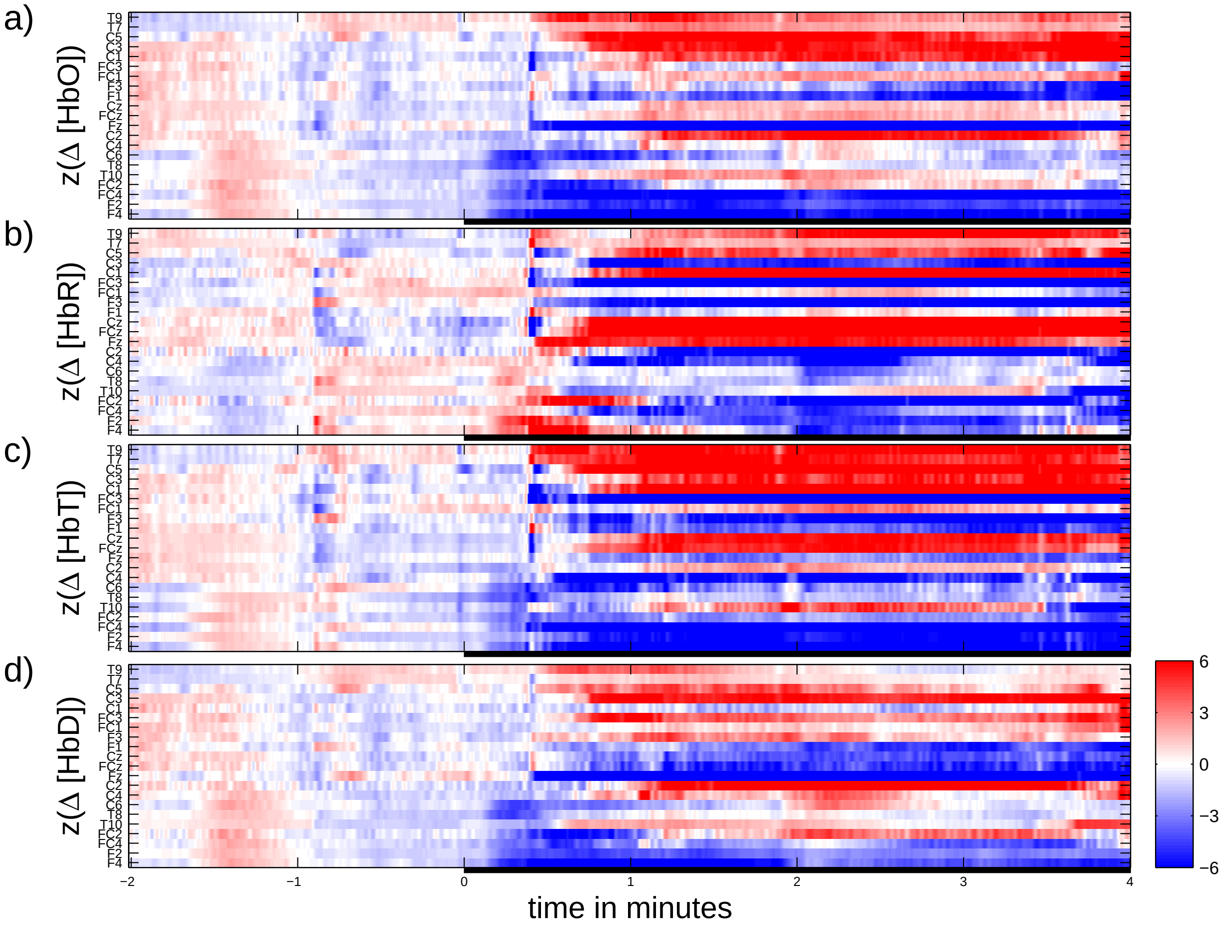

Figure S18: **Patient L2:** z-scores of all movement artifact corrected NIRS signals with respect to the last 120 seconds before prolonged occlusion of the right ICA (black bars on x-axes). The figure arrangement is identical to Fig. S6. This patient became neurologically symptomatic (transient dysarthria and left hemiparesis) and BOT was aborted.

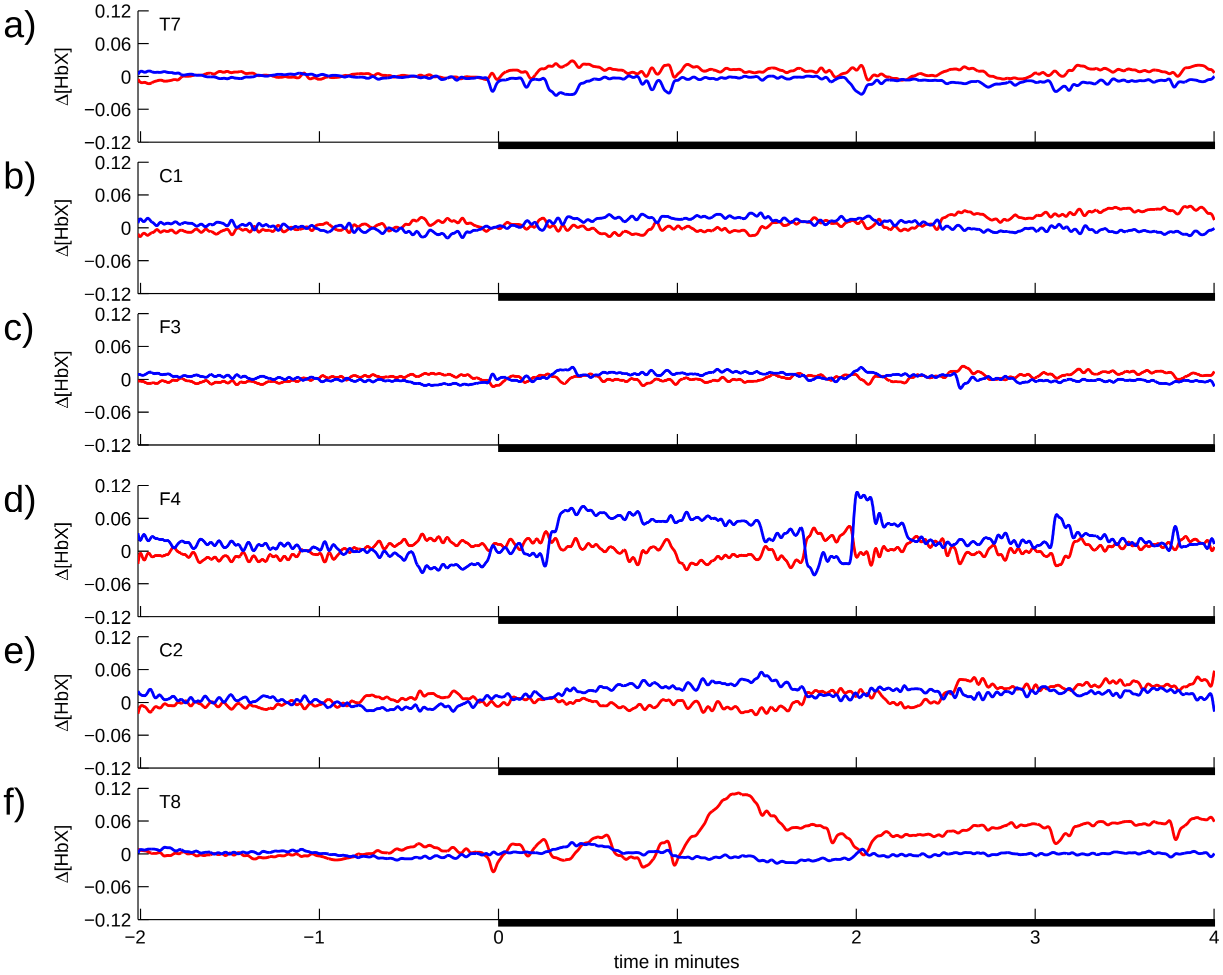

Figure S19: **Patient L3:** Selected NIRS time series during the first minutes of prolonged occlusion of the right ICA (black bars on x-axes). The figure arrangement is as in Fig. S5. Uncompensated movement artifacts (language and motor tasks during repeated neuropsychological testing) are visible on some NIRS signals approximately 10 seconds, 1 minute, 2 minutes, 3:05 minutes and 3:50 minutes after occlusion start.

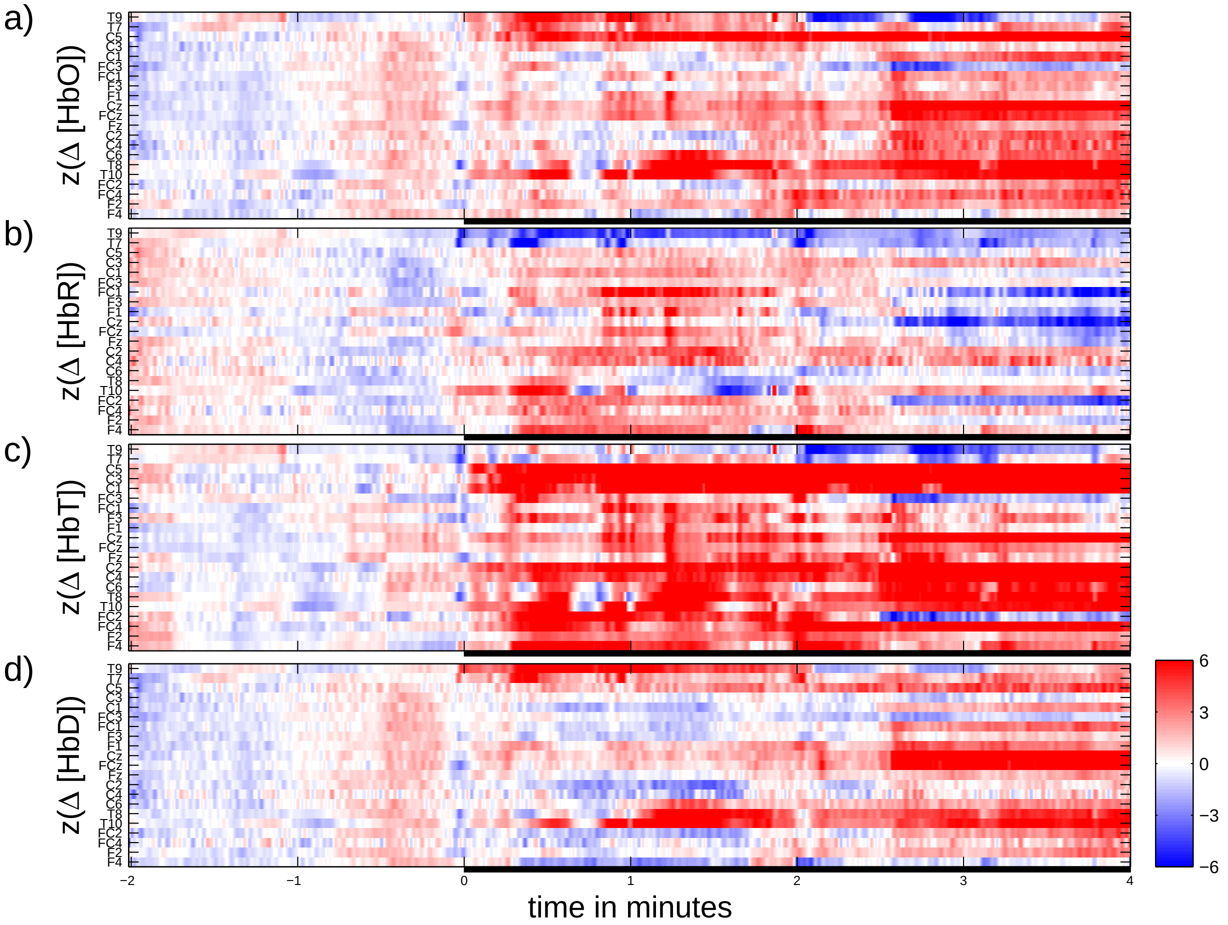

Figure S20: **Patient L3:** z-scores of all NIRS signals with respect to the last 120 seconds before prolonged occlusion of the right ICA (black bars on x-axes). The figure arrangement is identical to Fig. S6. Repeated neuropsychological testing showed no symptoms in this patient and contrast bolus injection revealed good collateralization.

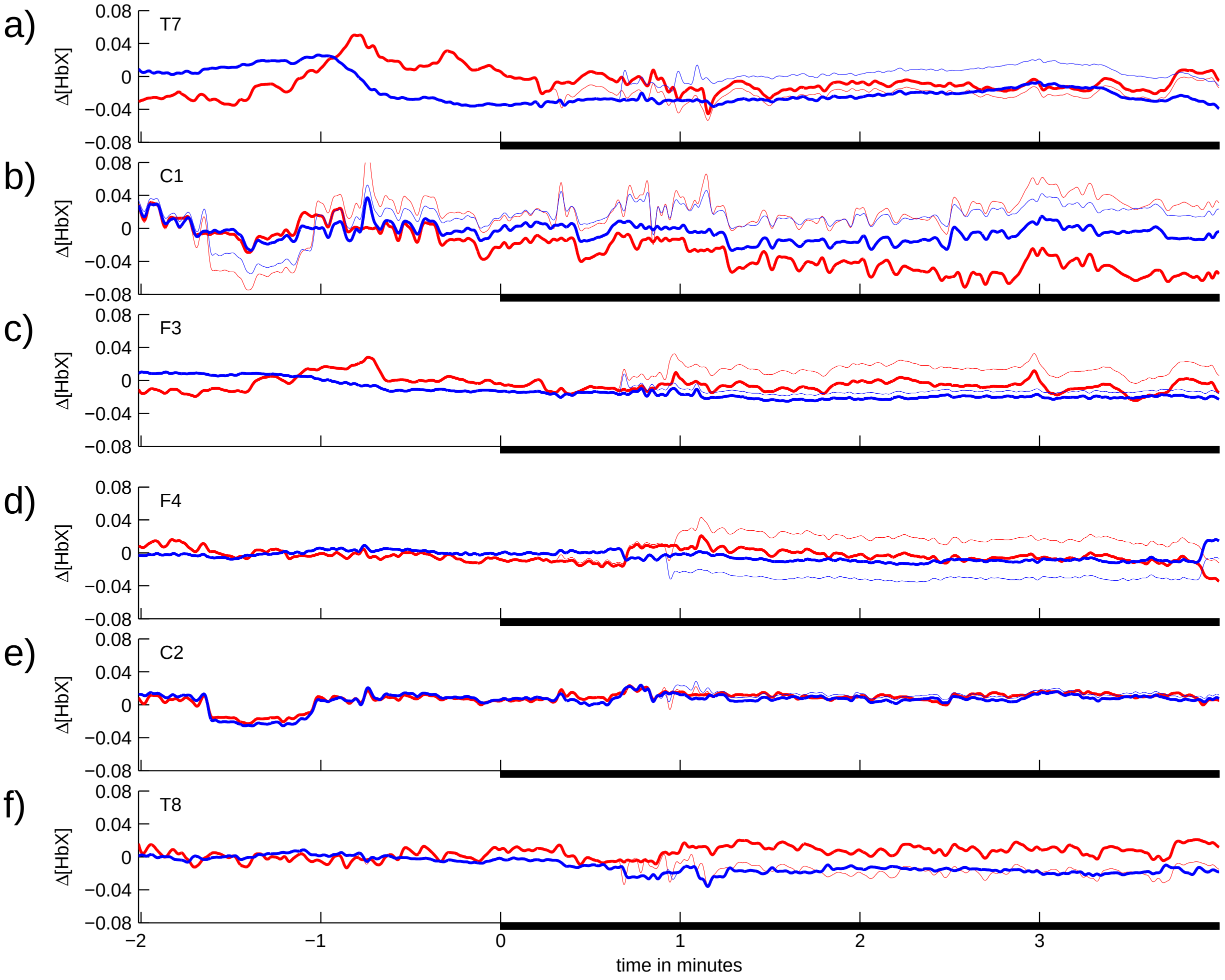

Figure S21: **Patient L4:** Selected NIRS time series during the first minutes of prolonged occlusion of the left ICA (black bars on x-axes). The figure arrangement is as in Fig. S5. Partly uncompensated movement artifacts are visible on some NIRS signals between 1 and 2 minutes before and approximately 1 minute after occlusion start.

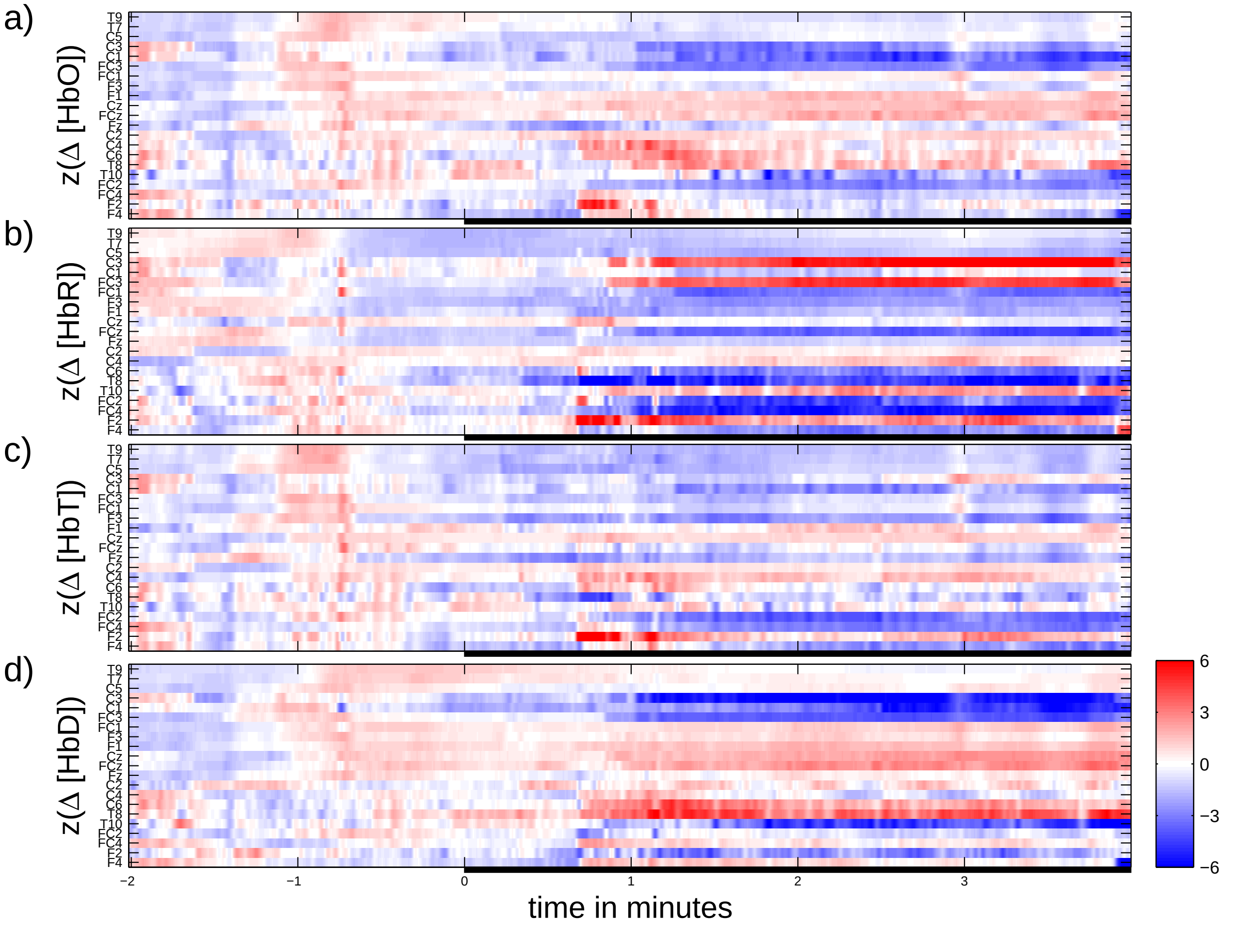

Figure S22: **Patient L4:** z-scores of all movement artifact corrected NIRS signals with respect to the last 120 seconds before prolonged occlusion of the left ICA (black bars on x-axes). The figure arrangement is identical to Fig. S6. Repeated neuropsychological testing showed no symptoms in this patient and contrast bolus injection showed good collateralization.

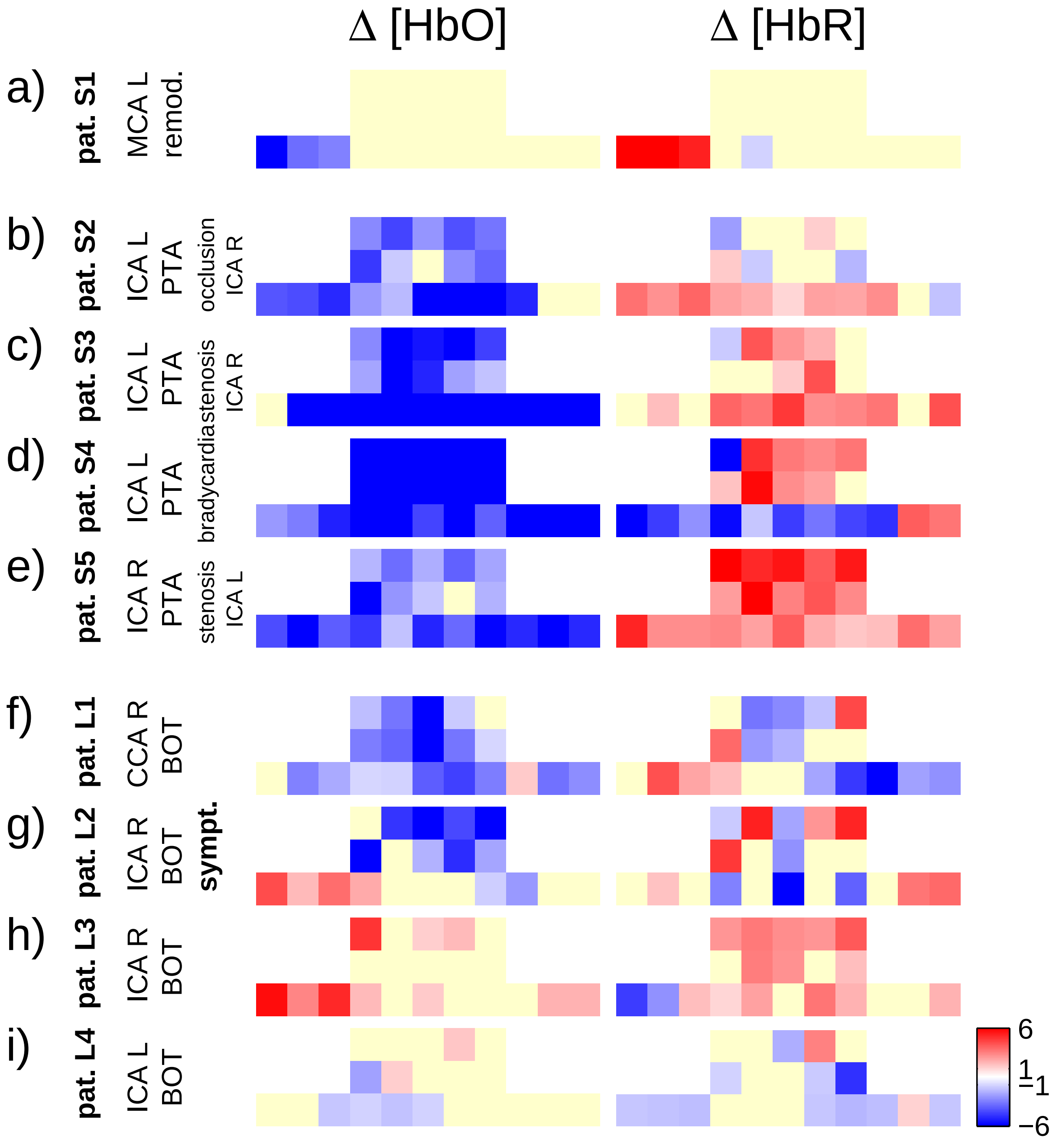

Figure S23: Spatial distribution of mean z-scores of Δ[HbO] and Δ[HbR] in 20s<t<60s (or the last 2/3 of shorter occlusions) for all patients. The spatial layout is the same as introduced in Fig. 3c of the main text. Channels where $-1 < \langle z \rangle < 1$ are indicated in light yellow. For patient S1 the mean z-score over all 20 vessel occlusions is shown.
